# Supplementary material for: Deep learning suggests that gene expression is encoded in all parts of a co-evolving interacting gene regulatory structure
Source: Nat Commun. 2020 Dec 1;11:6141. doi: 10.1038/s41467-020-19921-4 (PMC7708451; doi:10.1038/s41467-020-19921-4)
Supplement: Supplementary file 1 — Supplementary Information [file 41467_2020_19921_MOESM1_ESM.pdf]

Deep learning suggests that gene expression is encoded in all parts of a co-evolving interacting gene regulatory structure

Jan Zrimec[1], Christoph S. Börlin[1,5], Filip Buric[1], Azam Sheikh Muhammad[2], Rhongzen Chen[2], Verena Siewers[1,5], Vilhelm Verendel[2], Jens Nielsen[1,5], Mats Töpel[3,4], Aleksej Zelezniak[1,6]*

1 - Department of Biology and Biological Engineering, Chalmers University of Technology, Kemivägen 10, SE-412 96, Gothenburg, Sweden

2 - Computer Science and Engineering, Chalmers University of Technology, Kemivägen 10, SE-412 96, Gothenburg, Sweden

3 - Department of Marine Sciences, University of Gothenburg, Box 461, SE-405 30, Gothenburg, Sweden

4 - Gothenburg Global Biodiversity Center (GGBC), Box 461, 40530 Gothenburg, Sweden

5 - Novo Nordisk Foundation Center for Biosustainability, Chalmers University of Technology, Kemivägen 10, SE-412 96, Gothenburg, Sweden

6 - Science for Life Laboratory, Tomtebodavägen 23a, SE-171 65, Stockholm, Sweden

* corresponding author (email: aleksej.zelezniak@chalmers.se)

Table of contents

Supplementary figures	…	p.2 - p.41
Supplementary tables	…	p.42 - p.61
Supplementary references	…	p.62 - p.64

Supplementary figures

a.

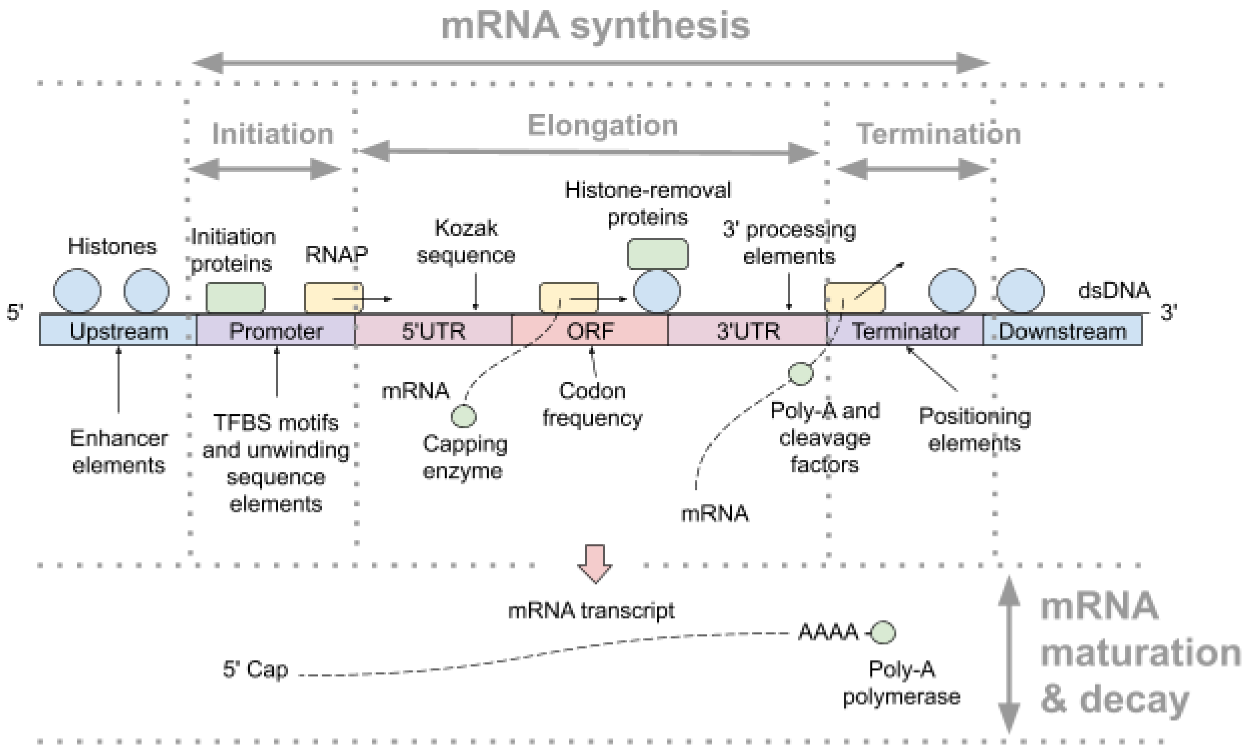

b.

Region	Promoter	5'UTR	Gene (CDS)	3'UTR	Terminator
R^2	0.46[a]	0.52[b]	0.55[c]	>0.16[d]	

[a] Not genome-wide [1]

[b] Expanded to 0.62 with deep learning [2,3]

[c] Target variable was mRNA half-life, up to 0.59 achieved with extra features [4]

[d] Estimated here based on multiple studies [5,6]

c.

Region	Promoter	5'UTR	CDS	3'UTR	Terminator
**Regulatory signals**	- Core promoter [7]<br>- TFBS [8]<br>- enhancers [9]	Kozak sequence [2,10]	Codon usage [11,12]	3' processing elements:<br>- A/T-rich sites [13]<br>- Positioning element [14]<br>- TA-rich efficiency el. [5]	
	Nucleosome positioning [6,12,15]				
**Size**	1,000 bp	300 bp	~300-3,000 bp	350 bp	500 bp
**Positioning**	to TSS	to START [2]	whole	to TTS [13]	from TTS
**Data types**	sequence	sequence, variables (2)	variables (67)	sequence, variables (2)	sequence
**Sequence data**	yes	yes	no	yes	yes
**Variable types**	/	length, GC content [4]	codon freq., length, GC of each wobble pos. [1,16]	length, GC content [4]	/

Supplementary figure 1. Schematic overview of published knowledge on the gene regulatory structure in *Saccharomyces cerevisiae*. (a) The molecular processes: schematic diagram of mRNA transcription in eukaryotes, detailing separate optimized processes, that form a fine-tuned regulatory system which spans mRNA synthesis, maturation and decay [12]. (b) The information content: overview of the approximate amount of information on gene expression levels that is encoded in each separate region according to published studies. (c) The regulatory system: overview of the known regulatory signals that contain information on gene expression, as well as the sequence parameters and variables used to model and predict gene expression levels in the present study. UTR denotes untranslated regions, ORF open reading frame, CDS coding sequence, TFBS transcription factor binding sites, TSS transcription start site, TTS transcription termination site.

Pathway	Description	BH adjusted P-value
GO:0005975	carbohydrate metabolic process	5.9e-06
GO:0006091	generation of precursor metabolites and energy	2.1e-10
GO:0006520	cellular amino acid metabolic process	8.5e-07
GO:0006811	ion transport	1e-04
GO:0006865	amino acid transport	0.026
GO:0006979	response to oxidative stress	0.0021
GO:0008643	carbohydrate transport	0.014
GO:0009311	oligosaccharide metabolic process	0.002
GO:0032787	monocarboxylic acid metabolic process	5.4e-05
GO:0042221	response to chemical	0.0082
GO:0045333	cellular respiration	8.7e-08
GO:0055085	transmembrane transport	0.0065
GO:0055086	nucleobase-containing small molecule metabolic process	5.4e-05

Supplementary figure 2. Enrichment analysis of gene ontology terms [17,18] in the most variable genes across the entire range of biological conditions (relative standard deviation, *RSD* > 1).

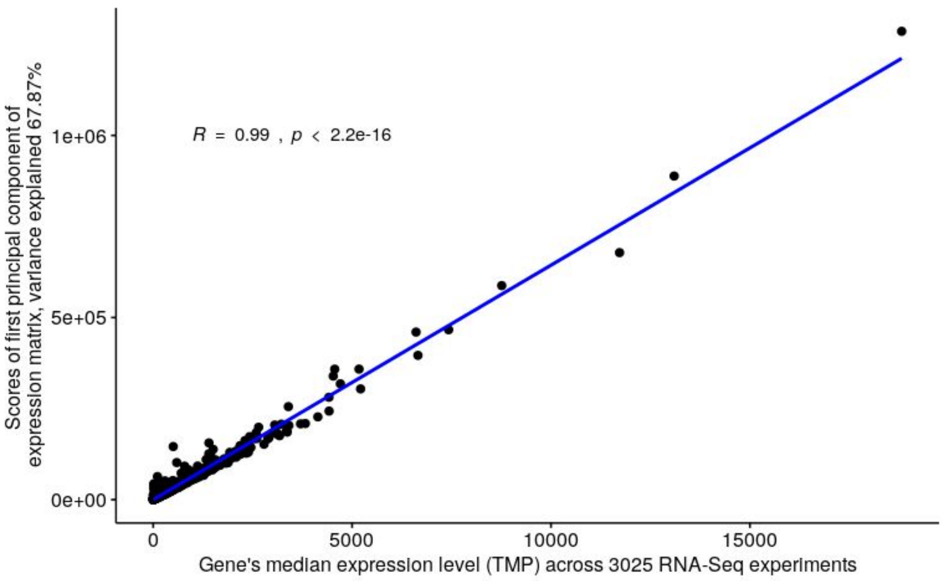

Supplementary figure 3. Median expression levels (transcripts per million, TPM) are representative of a gene's overall expression level across thousands of experiments, based on correlation analysis of the first principal component and median values of the entire matrix of mRNA counts (Pearson's r = 0.99, p-value < 2e-16, n = 4,238). Line denotes least squares fit.

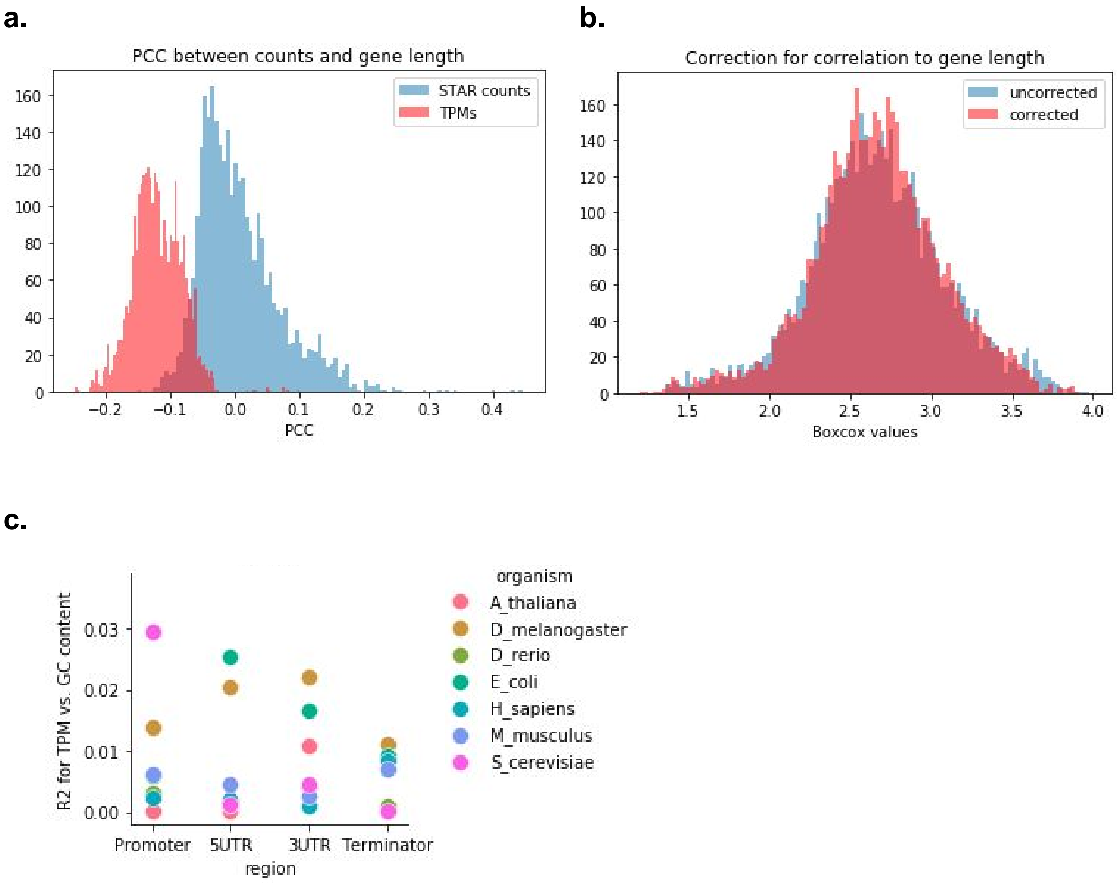

Supplementary figure 4. Overview for RNA-seq data processing with *Saccharomyces cerevisiae*. (a) A detectable level of correlation above 0.1 was observed between TPM (transcripts per million) transformed mRNA counts and gene (CDS) length. PCC denotes Pearson correlation coefficient. (b) Correction of the TPM target variable, by regressing out gene (CDS) length values, retained all information as the original uncorrected TPM values (Pearson's r = 0.96, p-value < 1e-16). (c) Overall GC content of regulatory regions was not predictive of gene expression levels, as the coefficient of determination (R^2) between gene expression values and GC content was below 3% for all model organisms. The different organisms are indicated by colors specified in the figure legend.

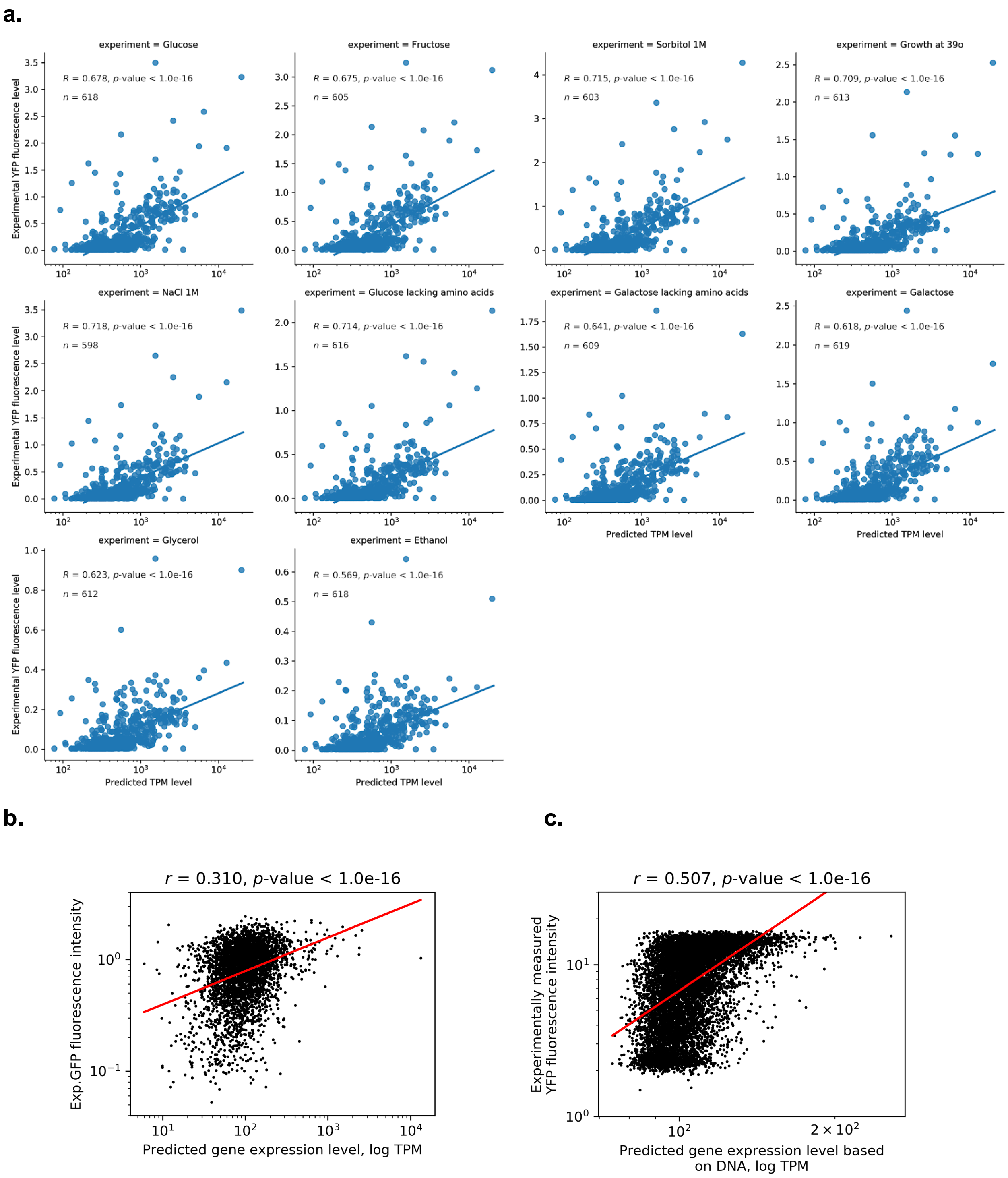

Supplementary figure 5. Model predictions are highly correlated with published experiments. (a) Experimental fluorescence measurements [19] versus predicted expression levels across 10 conditions by varying the promoter regions (see Results text). (b) Experimental fluorescence measurements [20] versus predicted expression levels by varying the terminators (n = 4,005, see Results text). (c) Experimental fluorescence measurements [21] versus predicted expression

levels on *de novo* sequence data comprising *n* = 9982 randomized promoter constructs within the ANP1 gene scaffold [21]. Model trained on *S. cerevisiae* data used in all analyses. All lines denote least squares fit, TPM transcripts per million.

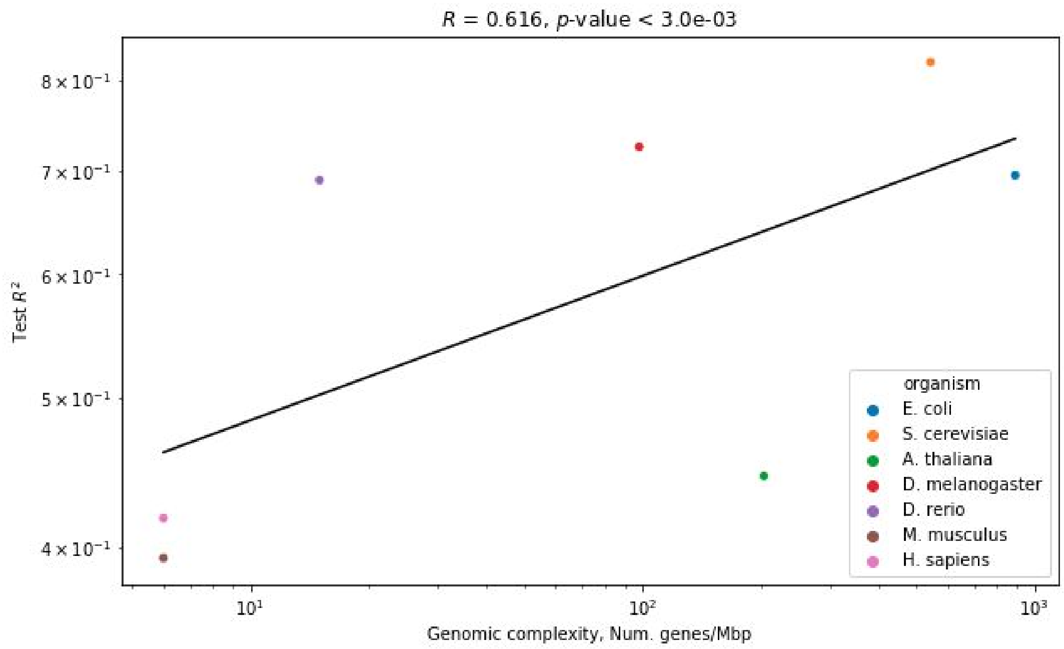

Supplementary figure 6. Correlation analysis between the predictive accuracy of deep learning models (R^2_{test}) and the genomic complexity across the model organisms (*n* = 7). Line denotes least squares fit. The different organisms are indicated by colors specified in the figure legend.

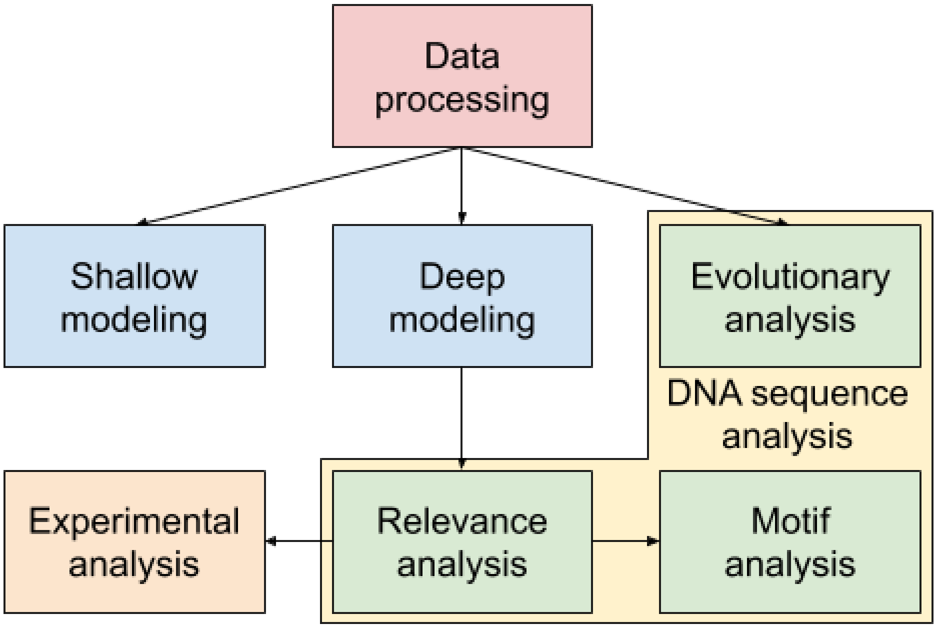

Supplementary figure 7. Overview of computational and experimental pipelines.

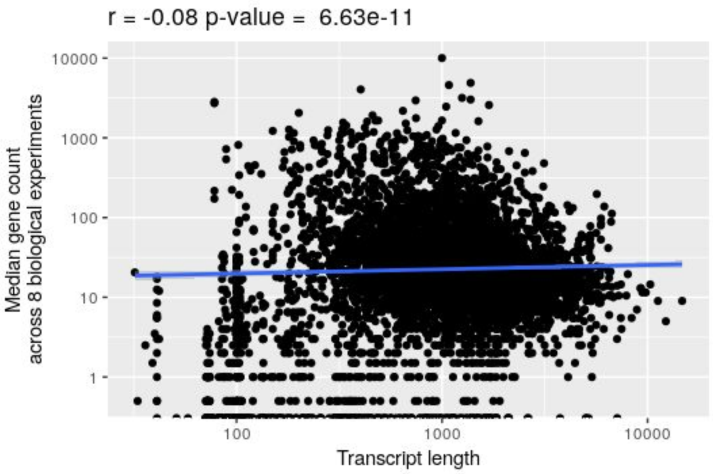

Supplementary figure 8. Correlation analysis between the gene length and the median expression level across experiments per gene, using data from whole molecule RNA-seq with the Oxford Nanopore MinION [22] (n = 6,486). Line denotes least squares fit.

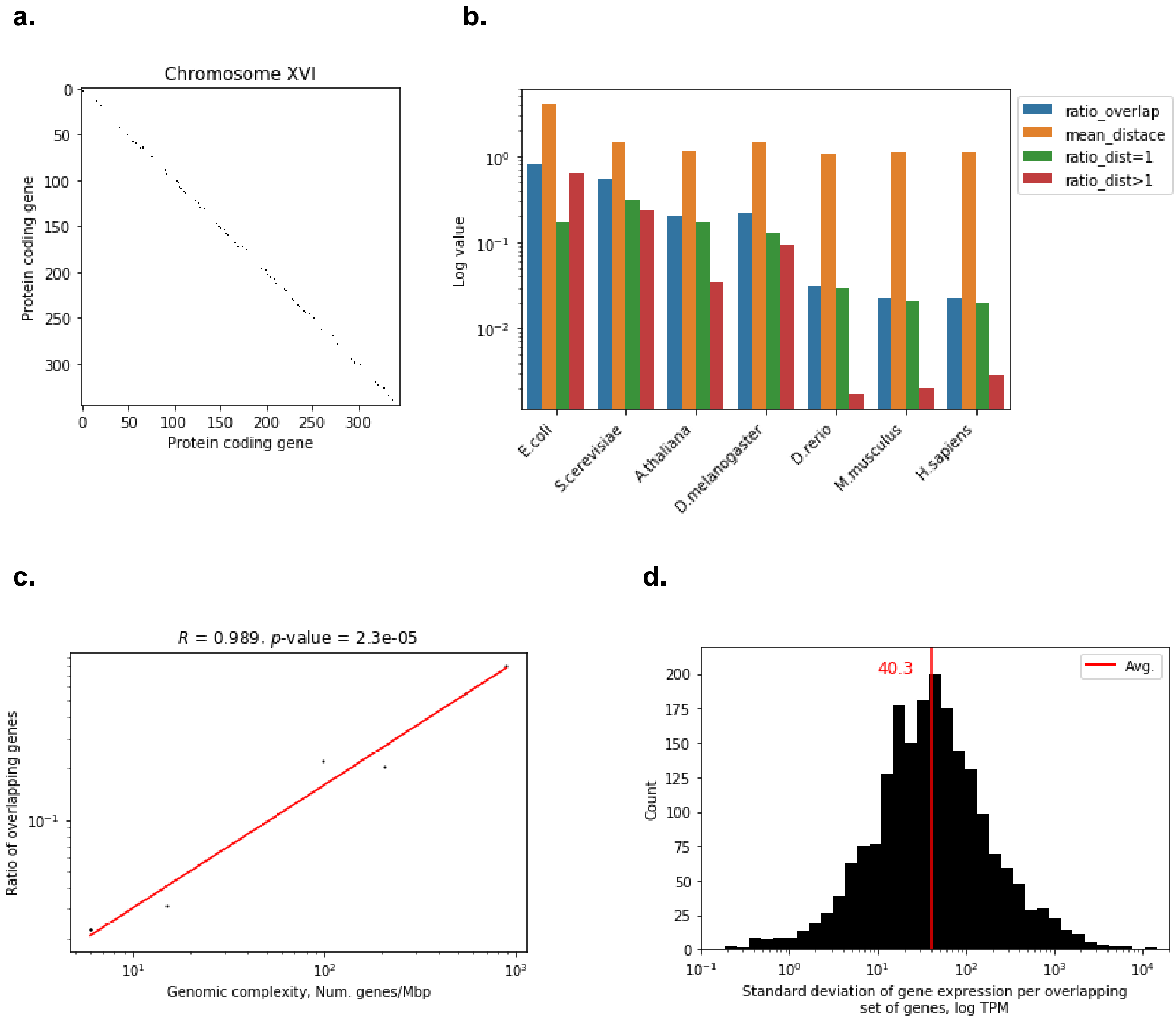

Supplementary figure 9. Analysis of overlap between the promoter and terminator regions of genes sorted according to the order of CDS occurrence. (a) Dot-plot of overlapping genes on chromosome 16 in yeast. Across all 16 yeast chromosomes, approximately half (55%) of genes promoters and terminators overlap with, on average, their first neighbor gene (80%), and up to 3 neighboring genes (20%) due to laying on opposing DNA strands. Mean gene overlap distance was 1.21. (b) Analysis of gene overlap across all 7 model organisms using the same boundaries (see Figure 1d) and metrics: the ratio of genes with overlaps out of all genes (ratio_overlap, blue), mean distance between overlapping genes (mean_distance, orange), ratio of genes overlapping with their nearest neighbor gene (ratio_dist=1, green) and ratio of genes overlapping with genes farther than their first nearest neighbor (ratio_dist>1, red). The number of overlapping genes rose to 80.7% for *E. coli* and fell to 2.2% with *H. sapiens*, with the mean distance of overlapping genes rising to 3.90 and falling to 1.01, respectively. (c) Correlation analysis between ratio of overlapping genes and genomic complexity (n = 7). As expected according to current knowledge [12,23,24], with increasing organism complexity (thus increasing

genome size and decreasing genomic complexity as measured by the number of genes per Mbp), the ratio of overlapping genes decreased, which was also the case with the other metrics. Lower complexity organisms, such as bacteria and yeast, have more compact genomes with less non-protein coding regions, and thus more overlap between regulatory elements [23,25] with less space for the more complex and distant regulation (e.g. enhancers that regulate gene expression from thousands of bps away) found in more complex organisms from plants to human [26–29]. (d) Variation of gene expression (median transcripts per million, TPM) observed across the overlapping sets of genes in yeast, with a median standard deviation of 40.3 TPM that reached a maximum of 12,479 TPM. This showed that, despite the overlaps in the regulatory regions and consequently a sharing of regulatory signals between some of the genes, expression levels between sets of overlapping genes can be highly variable.

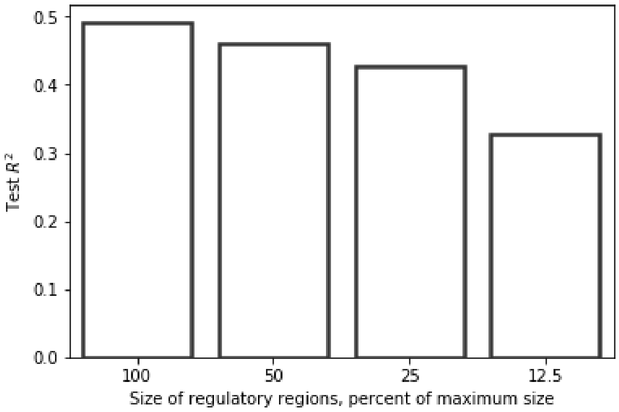

Supplementary figure 10. Analysis of the effect of decreasing the regulatory sequence sizes on model performance. All regions were anchored according to the sites in Figure 1d and the maximum sizes as defined in Figure 1c were used.

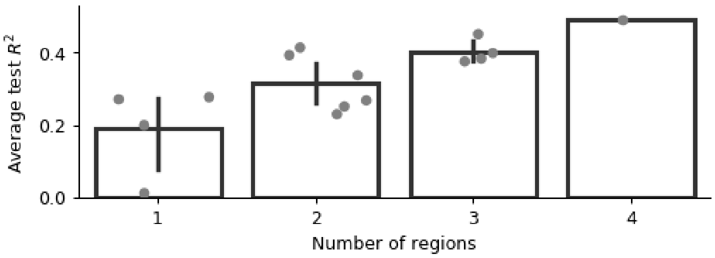

Supplementary figure 11. Effect of combinations of *cis*-regulatory regions on prediction of gene expression levels. Shown are the mean value and 95% confidence intervals of R^2_{test} at different amounts of regulatory regions used for training and testing the models (*n* = 4, 6, 4, 1, respectively).

a.

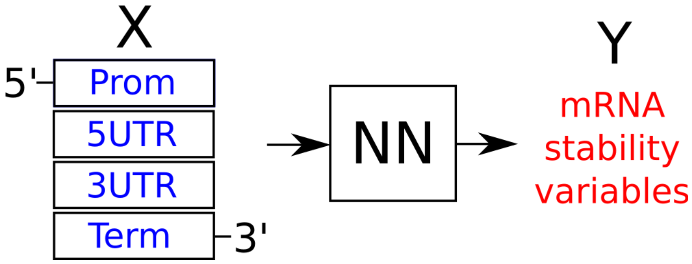

b.

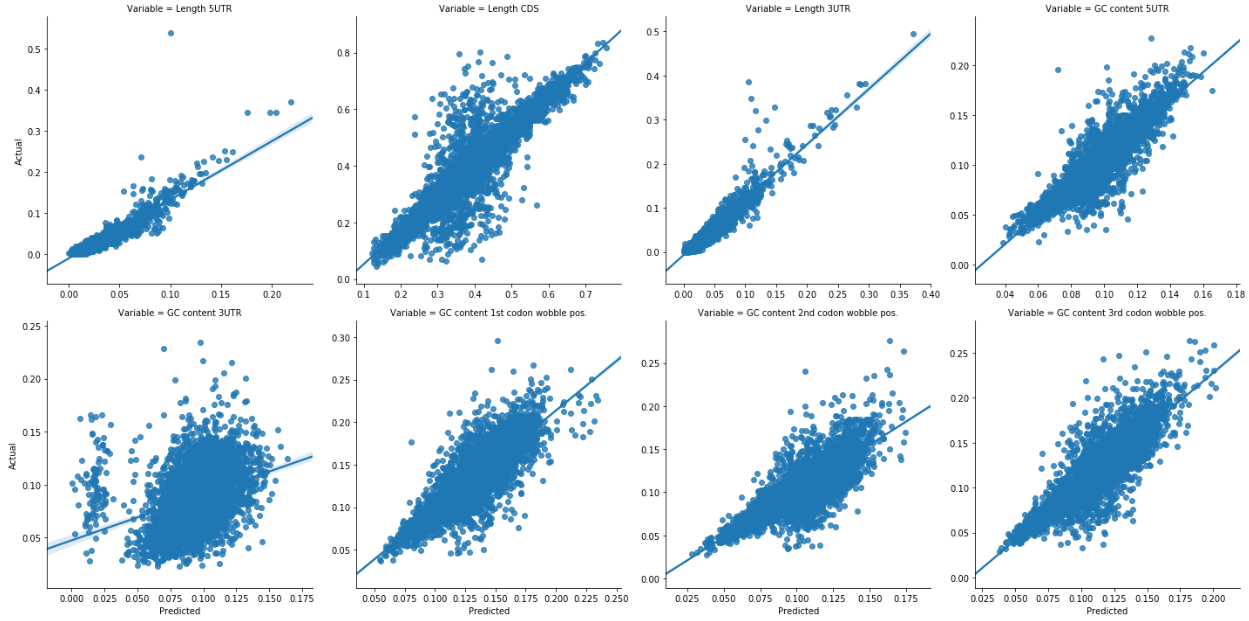

Supplementary figure 12. A CNN was built (a) that could predict nearly 80% of the variation of mRNA stability variables based on input regulatory sequences (R^2_{test} = 0.78). (b) Plots of actual versus predicted stability variables are shown, with individual R^2_{test} values of 0.788, 0.782, 0.864, 0.738, 0.146, 0.682, 0.645 and 0.684, respectively (*n* = 4,238 in all analyses). All lines denote least squares fit.

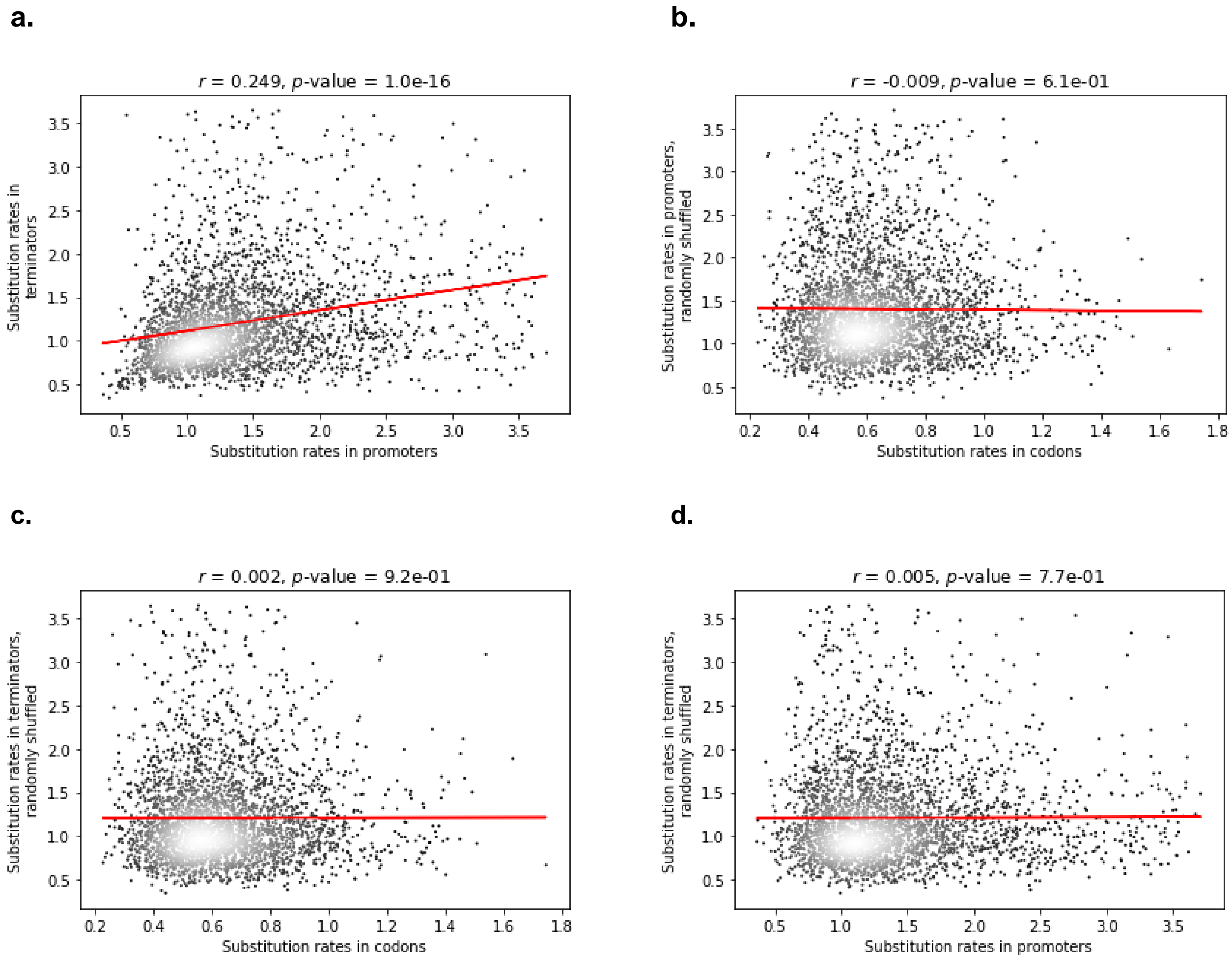

Supplementary figure 13. Analysis of coevolution of regulatory and coding regions in orthologous genes of 14 yeast species (n = 3,240 in all analyses). Red lines denote least squares fits. (a) Evolutionary substitution rates in terminators vs. promoter regions. (b) Control analysis of evolutionary substitution rates in promoter vs. coding regions, where the regions were randomly mismatched. (c) Control analysis of evolutionary substitution rates in terminators vs. coding regions, where the regions were randomly mismatched. (d) Control analysis of evolutionary substitution rates in terminators vs. promoter regions, where the regions were randomly mismatched.

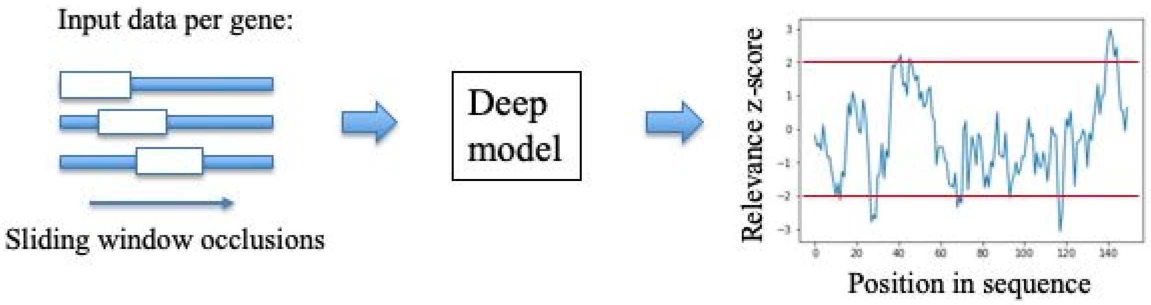

Supplementary figure 14. Schematic overview of the implemented DNA sequence occlusion-relevance approach [30,31].

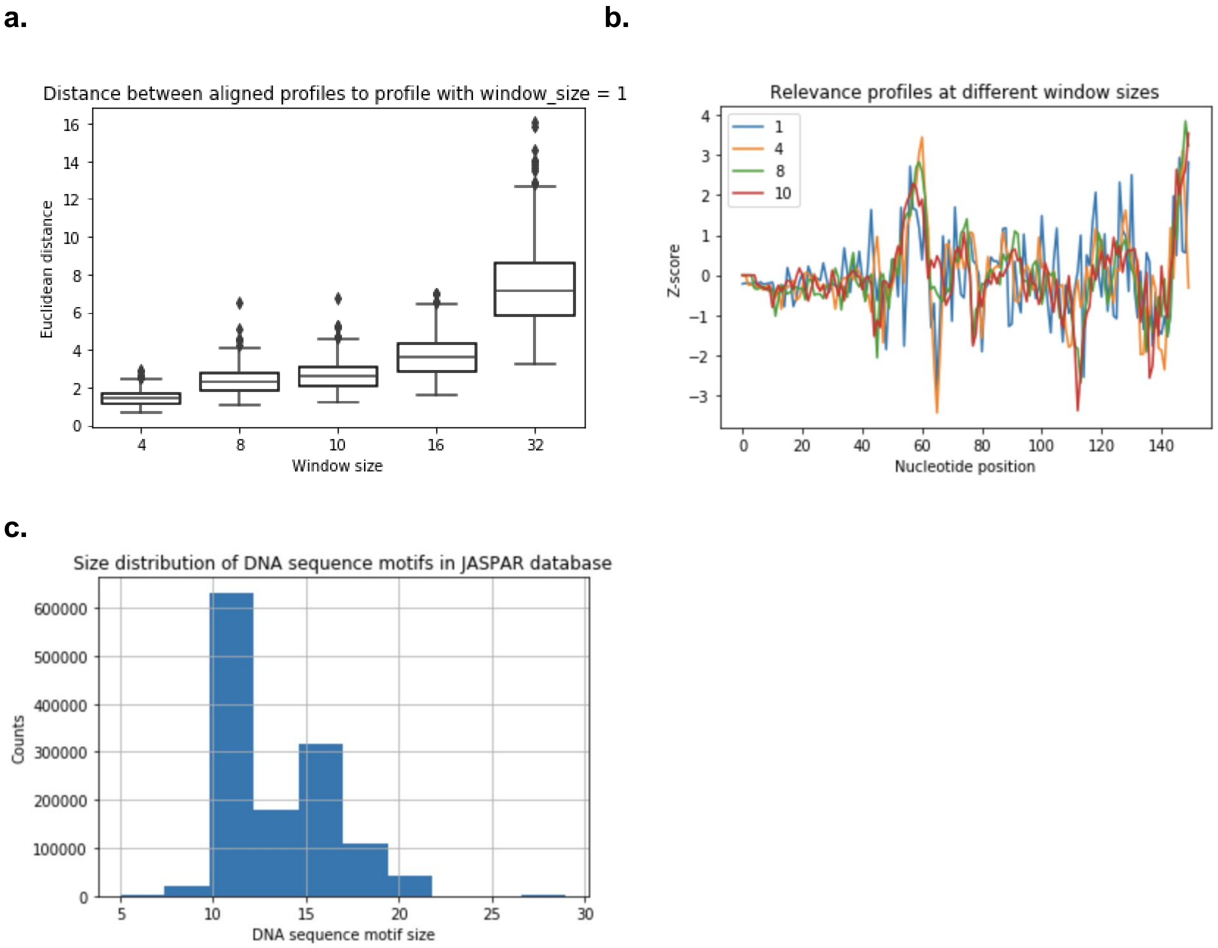

Supplementary figure 15. Analysis of different occlusion window sizes. (a) Euclidean distance between aligned profiles of sizes larger than 1 to the profiles with window size 1 (*n* = 425 with each window size). FastDTW alignment method used [32]. Boxes denote interquartile ranges (IQR), centres mark medians and whiskers extend to 1.5 IQR from the quartiles. (b) An example of the relevance profile with 150bps of a specific promoter region at different window sizes, which are indicated by colors specified in the figure legend. (c) Size distribution of DNA sequence motifs in JASPAR database (sites file: http://jaspar.genereg.net/download/sites.tar.gz). Considering that over 98% of DNA sequence motifs are 10 bps or larger, the analysis suggested that a window size of 10 was a good choice to recover the relevance of true DNA sequence motifs, whilst retaining the relevant information obtainable with the smaller window sizes.

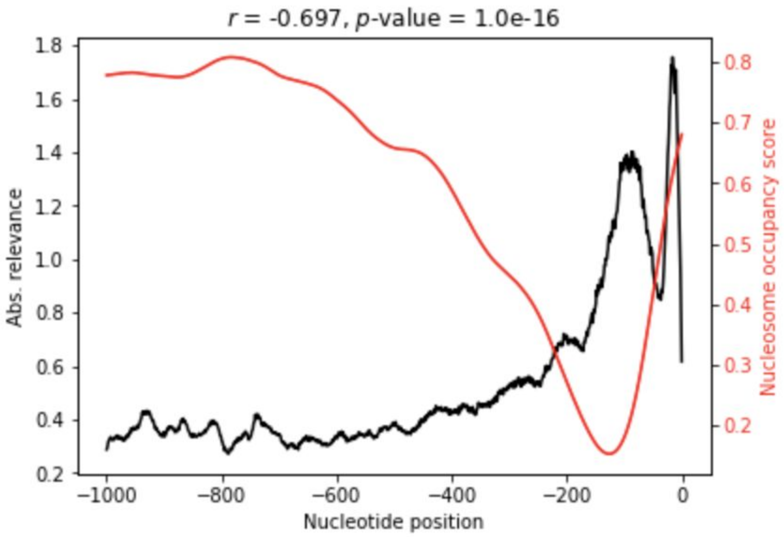

Supplementary figure 16. Strong correlation of absolute relevance in promoter regions (black) and published nucleosome occupancy scores [33] for TFIID regulated genes [34] (red), which were enriched (Fisher's exact test *p*-value < 1e-16) in the *S. cerevisiae* dataset.

Cluster	Pathway	Description	BH adjusted P-value
1	GO:0007059	chromosome segregation	1.9e-04
1	GO:0033043	regulation of organelle organization	4.4e-03
1	GO:0048285	organelle fission	4.5e-03
4	GO:0002181	cytoplasmic translation	0.0e+00
4	GO:0005975	carbohydrate metabolic process	8.1e-04
4	GO:0006091	generation of precursor metabolites and energy	3.5e-05
4	GO:0006414	translational elongation	3.1e-06
4	GO:0006520	cellular amino acid metabolic process	6.8e-05
4	GO:0032787	monocarboxylic acid metabolic process	7.0e-06
4	GO:0051186	cofactor metabolic process	6.9e-05
4	GO:0055086	nucleobase-containing small molecule metabolic process	1.0e-05

Supplementary figure 17. Enrichment analysis of gene ontology terms [17,18] in Cluster 4 (with high expressed genes) of the clustered relevance profiles.

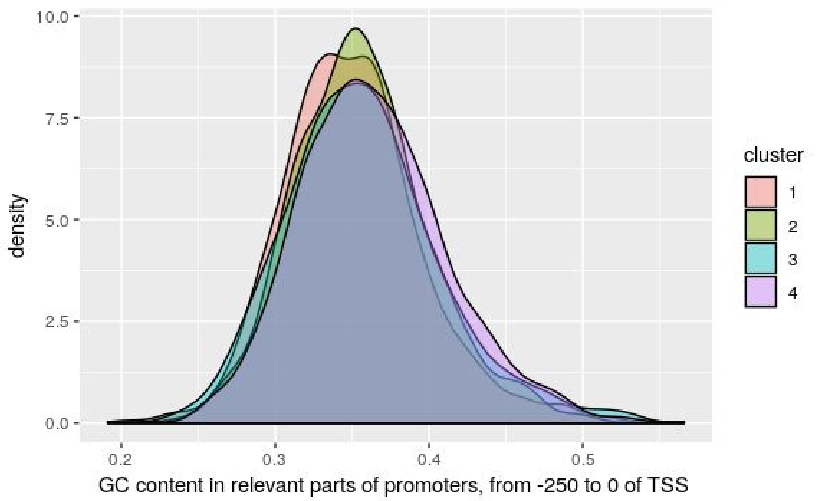

Supplementary figure 18. Clusters of relevance scores are independent of the DNA nucleotide composition. The different clusters are indicated by colors specified in the figure legend.

a.

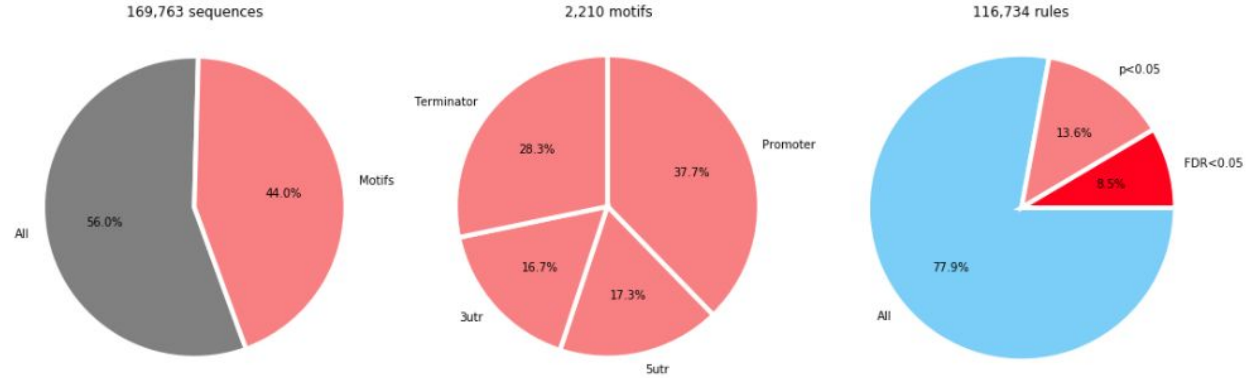

b.

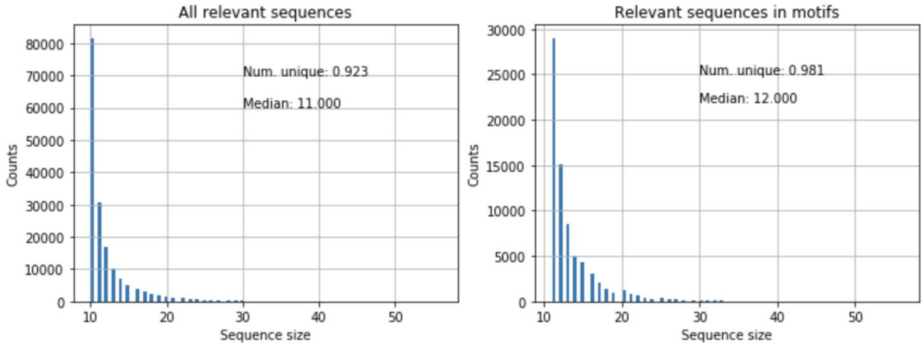

c.

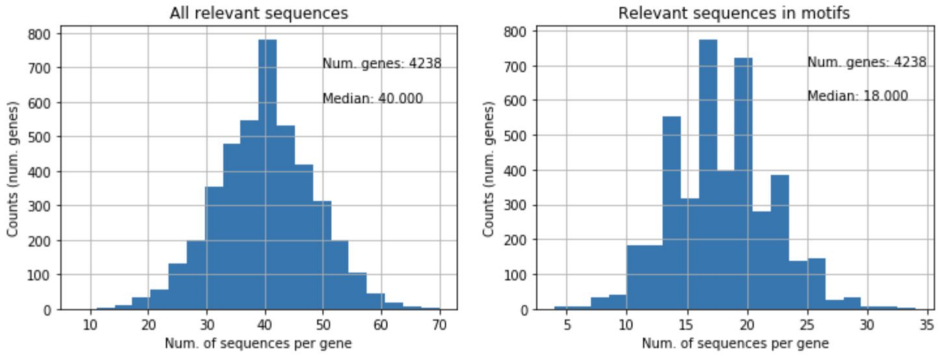

Supplementary figure 19. Analysis of significantly relevant DNA sequences. (a) 169,763 DNA sequences with significant relevance scores (exceeding 95% of range of values, i.e. ± 2 standard deviations) were extracted from the relevance profiles and used to construct regulatory DNA motifs and motif co-occurrence rules. Motif distributions across the *cis*-regulatory regions are shown. (b) Distribution of sizes of all relevant sequences and only those used for constructing the motifs (74,728 at 80% sequence identity cutoff, see Supplementary table 11). (c) Similarly, distribution of the amount of relevant sequences per gene showed good coverage of the whole set of genes with the extracted regulatory DNA motifs.

a.

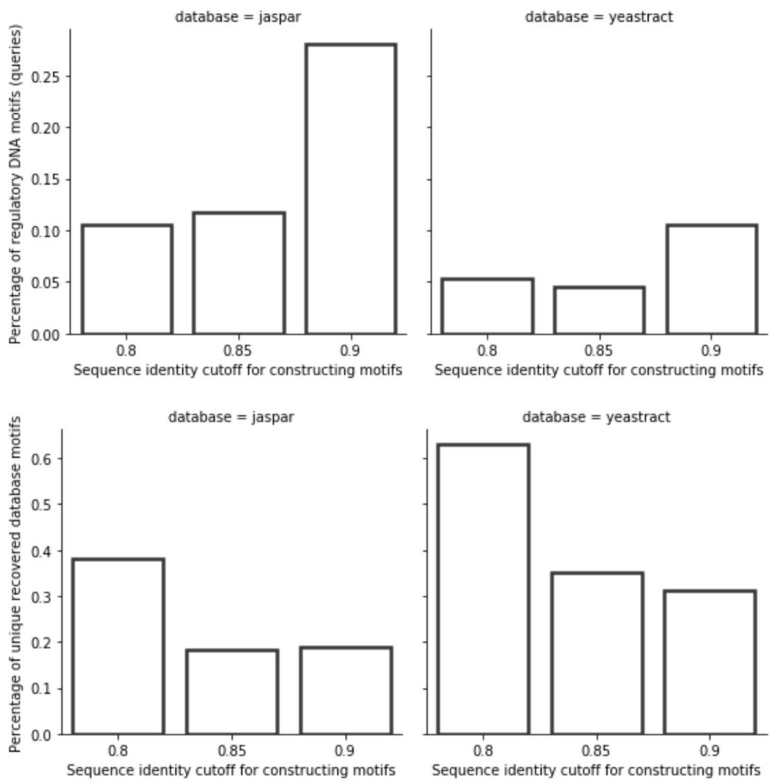

b.

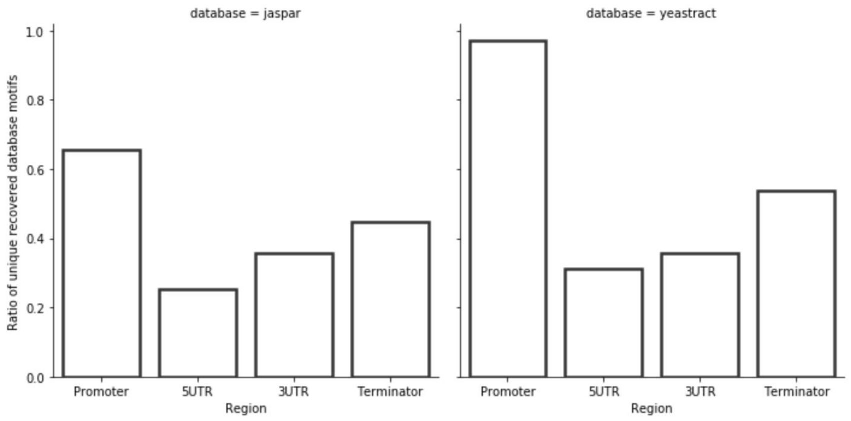

Supplementary figure 20. Comparison of constructed regulatory DNA motifs to JASPAR [35] and Yeastract [36] databases. (a) Although the number of regulatory DNA motifs that are significantly (BH adj. *p*-value < 0.05) similar to ones in databases increases with the increasing sequence identity cutoff used to construct the motifs, the number of unique recovered database motifs decreases, with the exception at the sequence identity cutoff of 0.85 with the Yeastract database. (b) Distribution of significant (BH adj. *p*-value < 0.05) motif hits from the JASPAR [35] and Yeastract [36] databases according to the regulatory regions, where the constructed regulatory motif queries were obtained.

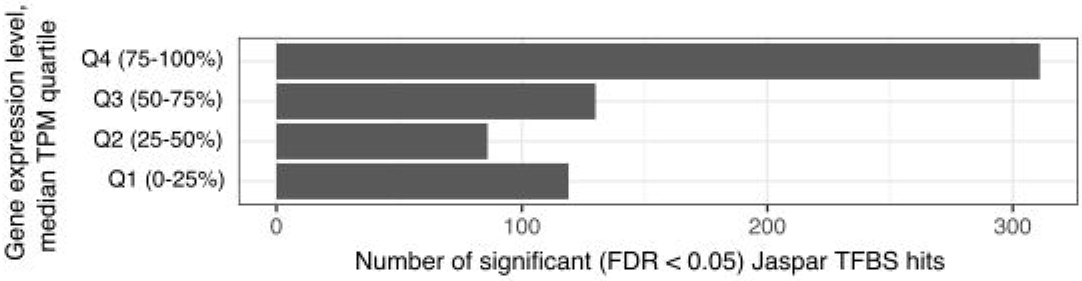

Supplementary figure 21. Enrichment of known yeast transcription factor binding sites (TFBS) from the Jaspar database [35] in promoters of *Saccharomyces cerevisiae* genes, which were binned into quartiles based on median expression levels (transcripts per million, TPM).

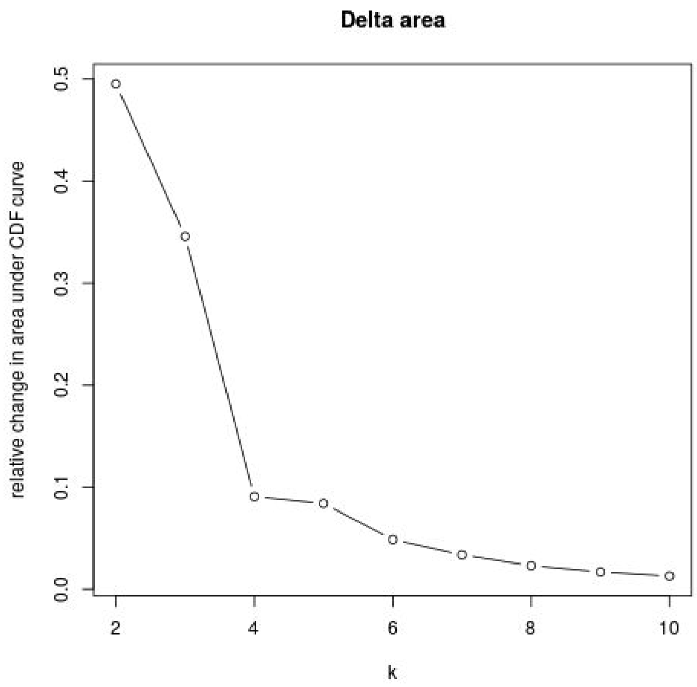

Supplementary figure 22. For clustering of relevance profiles the optimal amount of clusters *k* was determined at 4 (Methods).

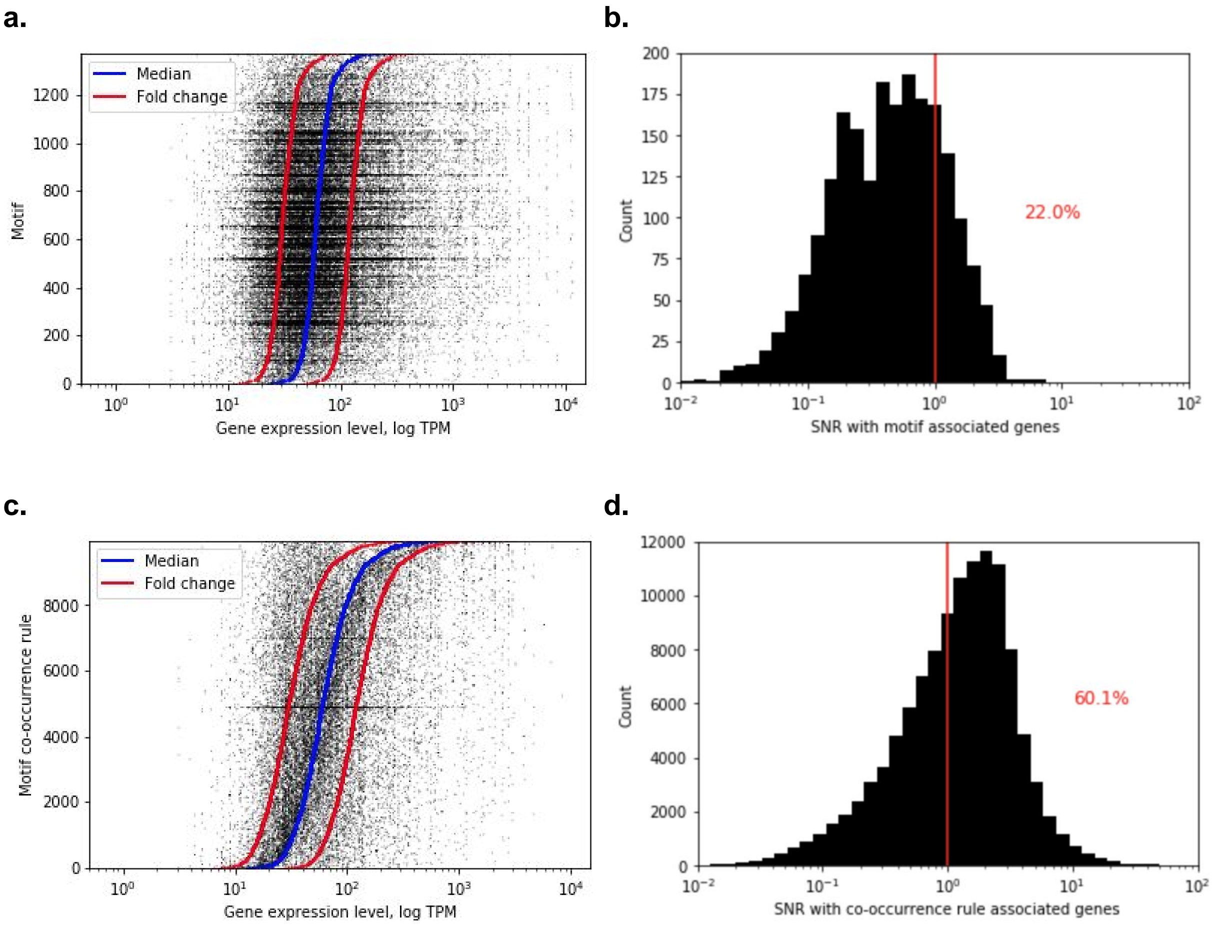

Supplementary figure 23. The range and precision of gene expression regulation with regulatory DNA motifs and motif co-occurrence rules. (a) Expression levels of genes associated with single motifs. (b) Distribution of the signal-to-noise ratio (*SNR*) of expression levels of genes associated with single motifs. Red line denotes an *SNR* of 1. (c) Expression levels of genes associated with motif co-occurrence rules. (d) Distribution of the signal-to-noise ratio (*SNR*) of expression levels of genes associated with motif co-occurrence rules. Red line denotes a *SNR* of 1, TPM transcripts per million.

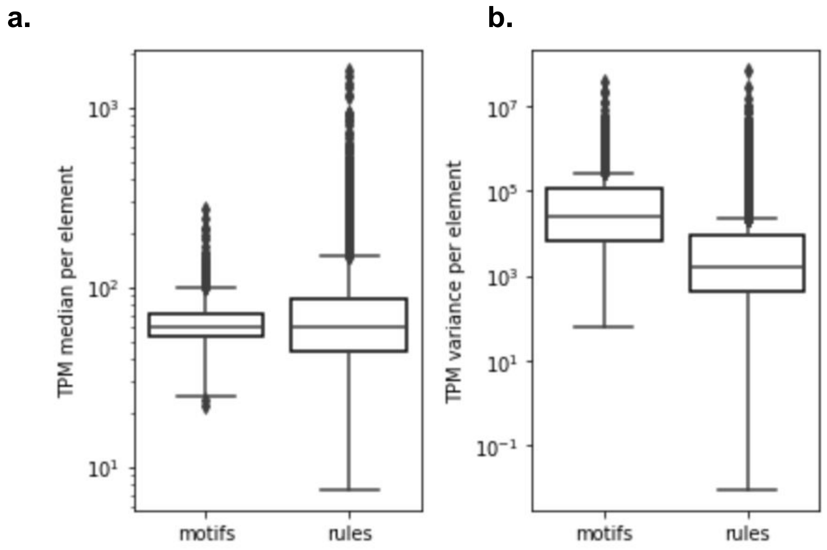

Supplementary figure 24. (a) Median and (b) variance of gene expression levels (transcripts per million, TPM) with genes associated with single regulatory DNA motifs (n = 1,374) or motif co-occurrence rules (n = 9,962). Boxes denote interquartile ranges (IQR), centres mark medians and whiskers extend to 1.5 IQR from the quartiles.

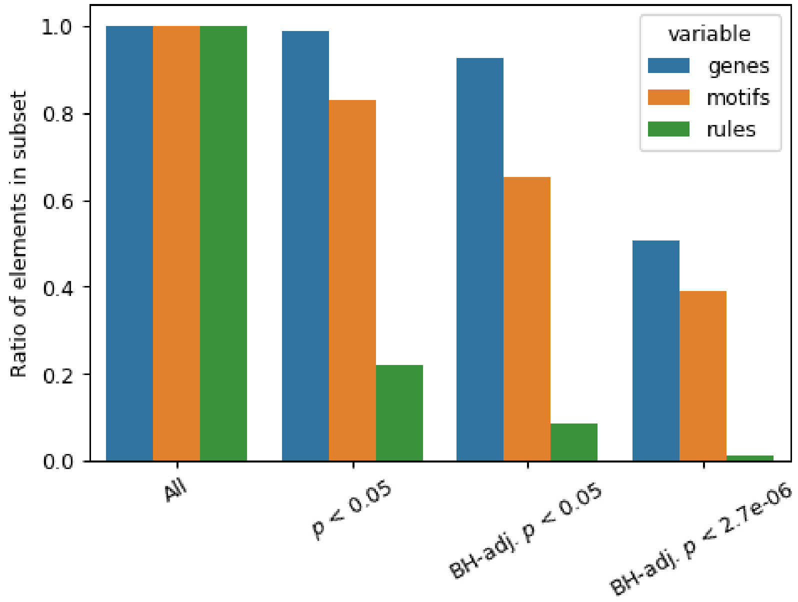

Supplementary figure 25. Ratio of retained elements: unique genes (blue), regulatory DNA motifs (orange) and motif co-occurrence rules (green), with increasing statistical stringency (chi-squared test [37], Benjamini-Hochberg, BH adjustment) (Figure 4b).

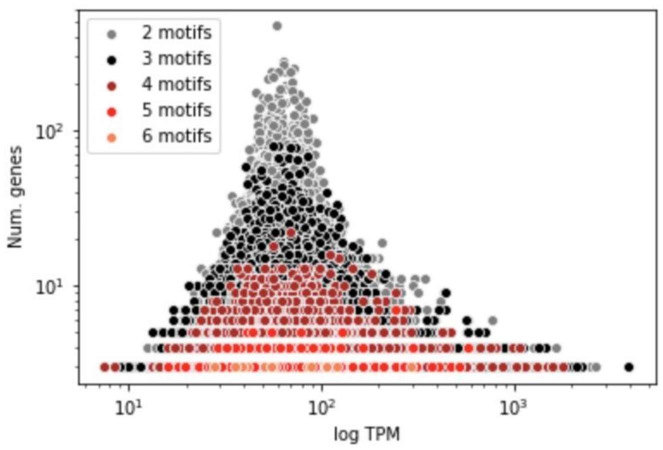

Supplementary figure 26. The amount of genes that carry a specific regulatory DNA motif co-occurrence rule versus the average expression level (transcripts per million, TPM) across the genes that carry the given rule. Data corresponding to the amount of co-occurring motifs per rule from 2 through 6 are colored grey, black, dark red, red and light red, respectively.

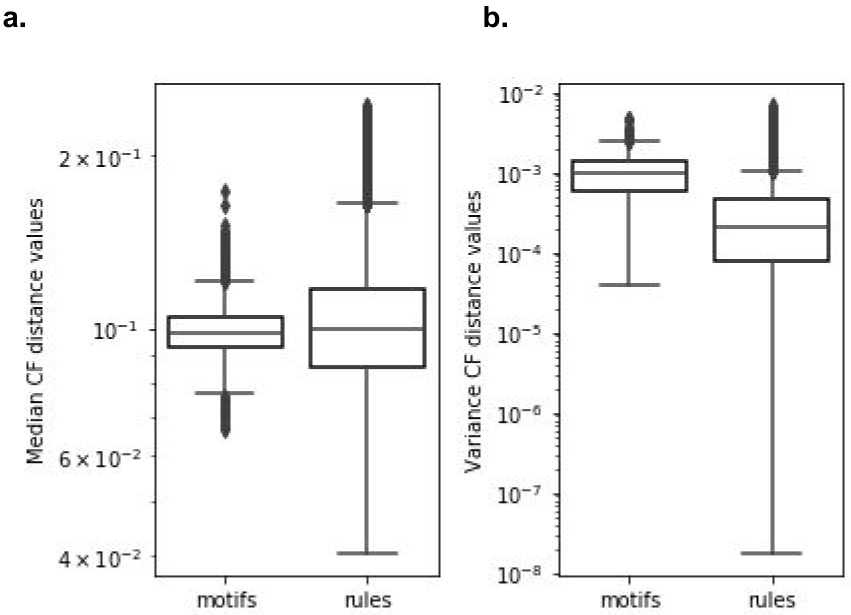

Supplementary figure 27. (a) Median and (b) variance of Euclidean distances between codon frequencies (CF) within genes defined by single regulatory DNA motifs (*n* = 1,374) or motif co-occurrence rules (*n* = 9,962). Boxes denote interquartile ranges (IQR), centres mark medians and whiskers extend to 1.5 IQR from the quartiles.

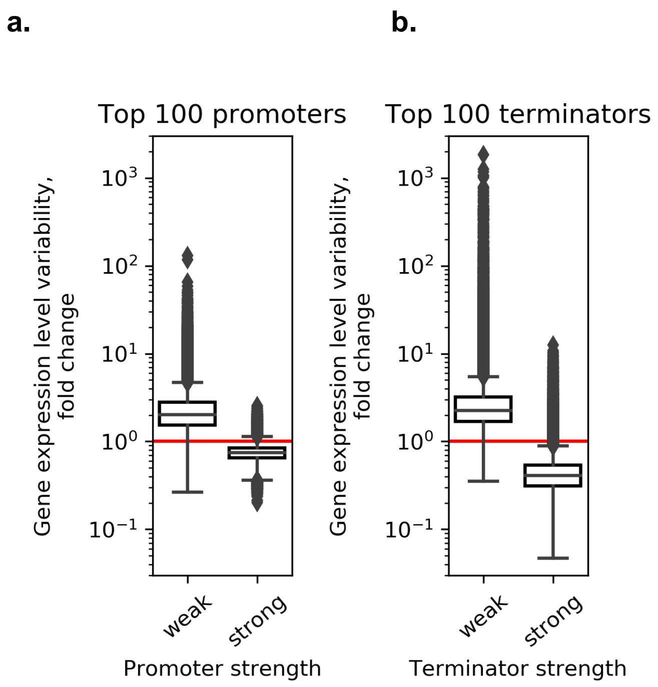

Supplementary figure 28. Variation of gene expression with strong and weak regulatory regions, represented by the selection of 100 top and bottom sorted constructs (*n* = 423,800 for each strength level). (a) Native promoters combined with different terminators. (b) Native terminators combined with different promoters. Boxes denote interquartile ranges (IQR), centres mark medians and whiskers extend to 1.5 IQR from the quartiles.

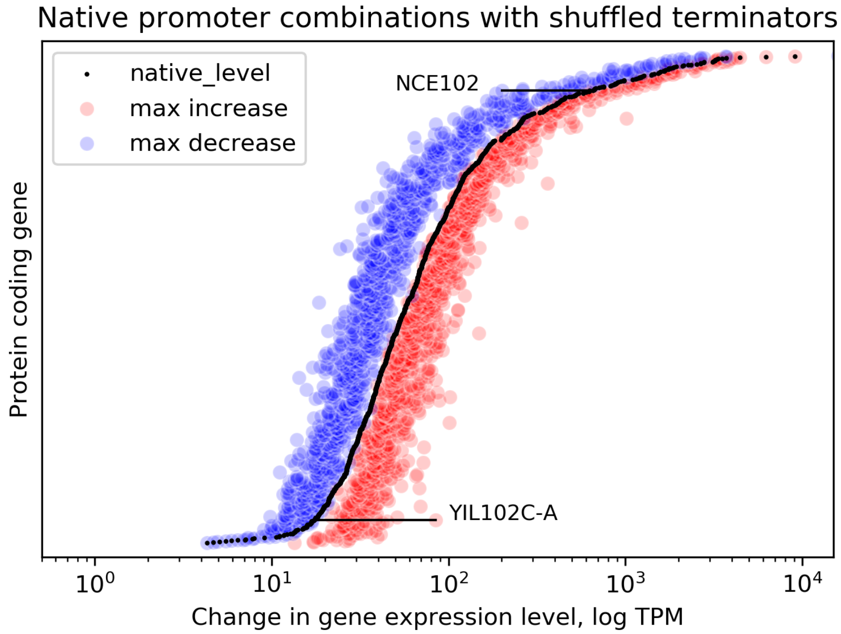

Supplementary figure 29. Evaluation of the effect of removing high-order sequence information (ie. regulatory grammar) by randomly shuffling the DNA in regulatory regions whilst preserving dinucleotide frequencies [38]. On average, these constructs achieved a 1.4 -fold change in either direction of expression levels (increases colored red, decreases blue, native levels black; transcripts per million, TPM) and a dynamic range below 1 order of magnitude (6.3 -fold range with YIL102C-A).

a.

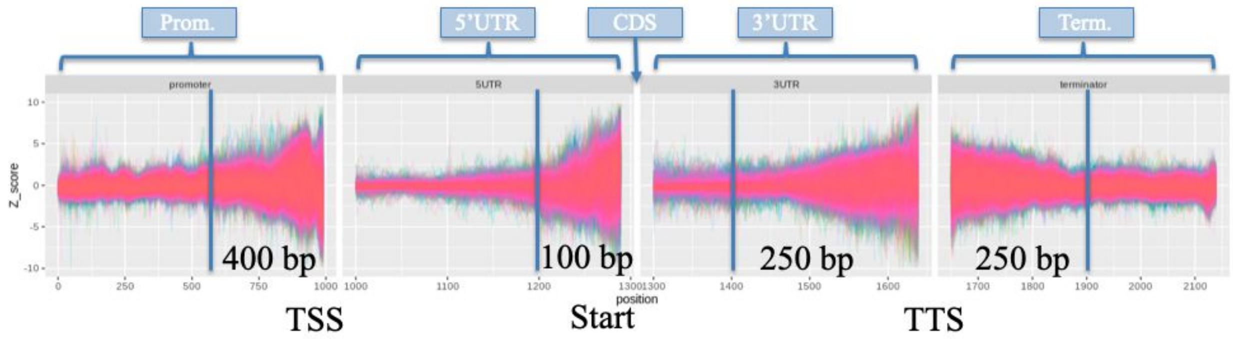

b.

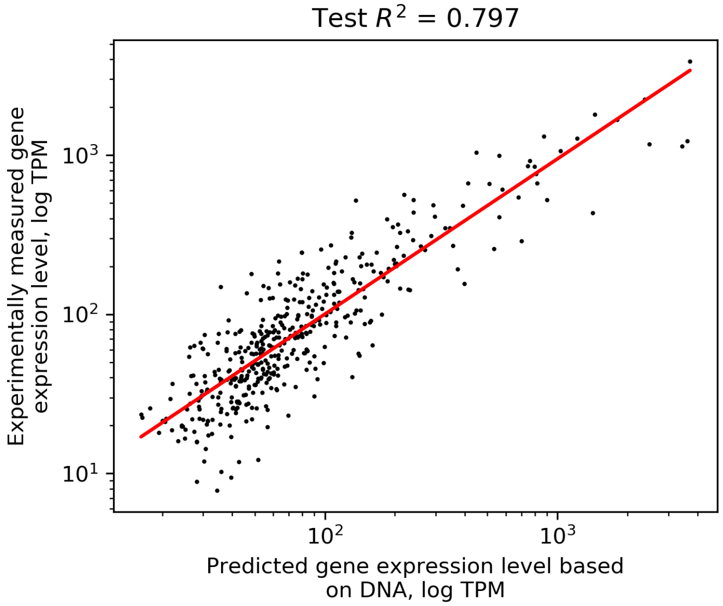

Supplementary figure 30. Construction of new input data and model based on altogether 1,000 bps of regulatory sequence (500 bp on each side of coding region), as required for experiments. (a) Relative relevance of input data across the different regions guided the selection of smaller regions parts with largest overall relevance. Cutoffs are marked with blue vertical lines and final region lengths are specified. (b) Experimentally determined versus predicted expression levels (transcripts per million, TPM) with the new model based on 1,000 bps of regulatory sequence (n = 425). Red line denotes least squares fit.

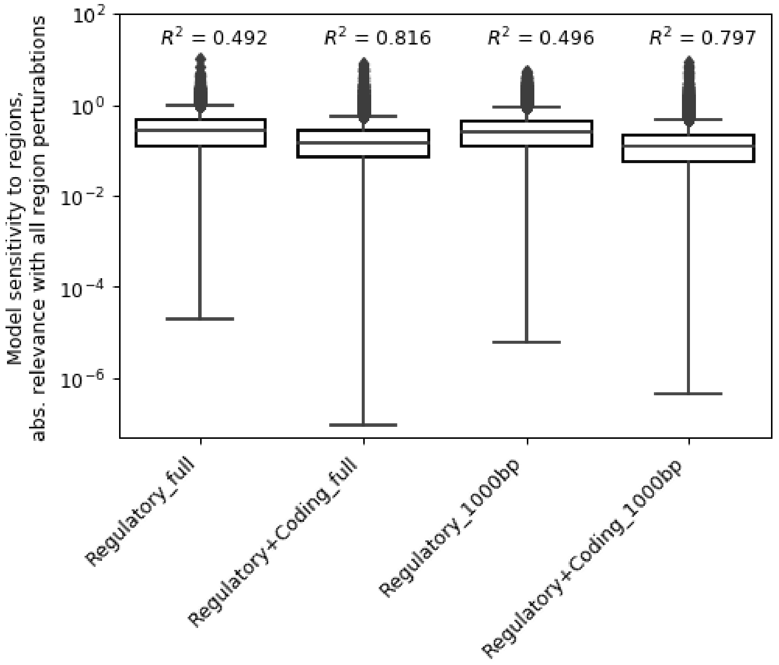

Supplementary figure 31. Analysis of the sensitivity of models to regulatory region perturbations. The sensitivity was assessed from the distribution of absolute relevance scores per model. Models based on either the full or 1,000 bp of regulatory sequence and with mere regulatory regions or in combination with coding regions are shown. Median values were 0.269, 0.151, 0.259 and 0.122, respectively, showing that with the new models based on merely 1,000 bps of regulatory sequence, apart from similar performance, also a similar model response was achieved. Boxes denote interquartile ranges (IQR), centres mark medians and whiskers extend to 1.5 IQR from the quartiles.

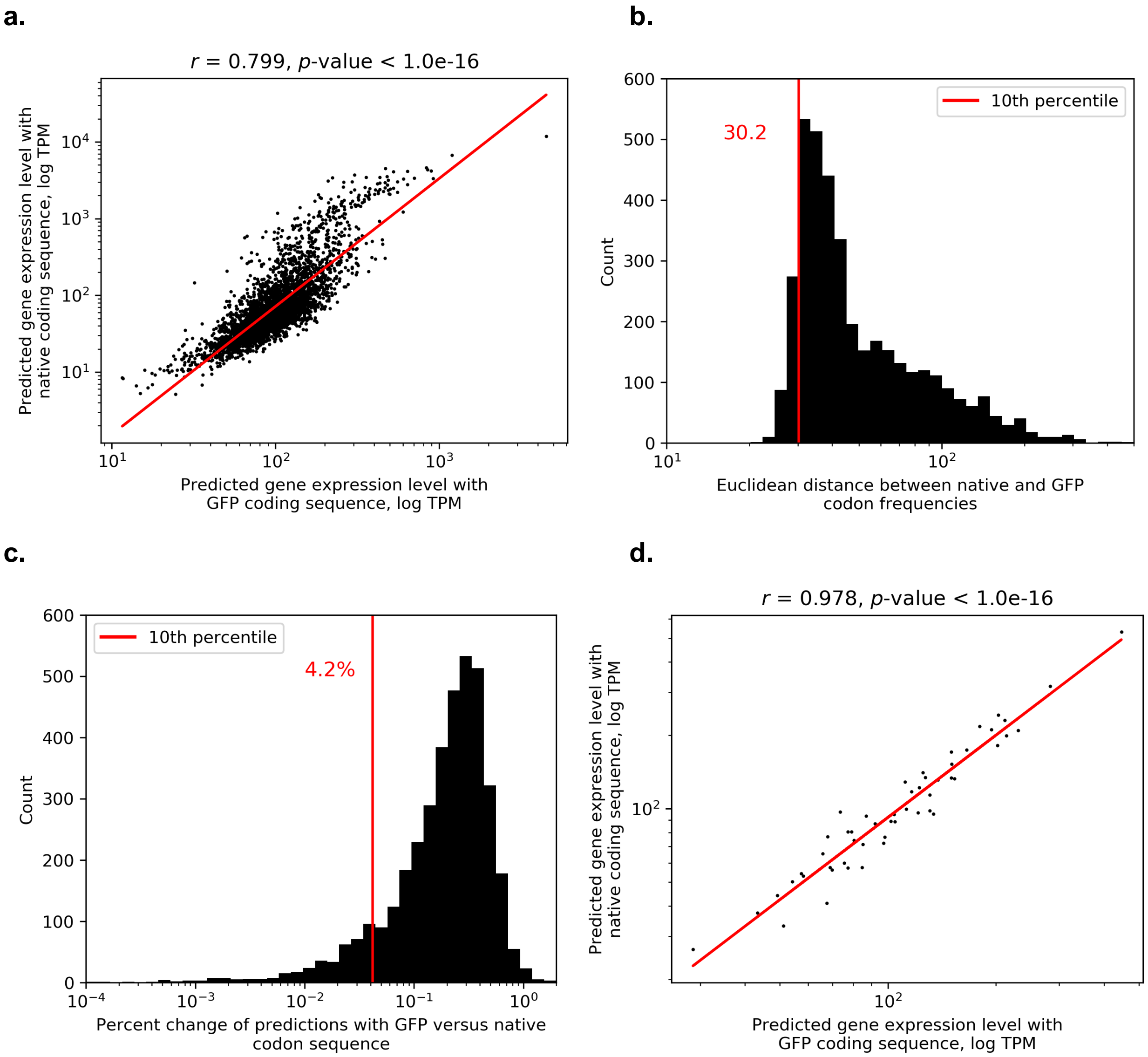

Supplementary figure 32. Selection of experimental constructs. (a) Correlation analysis of model predictions with perturbed input data with GFP codon frequencies versus native ones (*n* = 3,820). To select a subset of the data with more accurate model predictions with GFP-substituted coding sequences, both (b) the Euclidean distance between native and GFP codon frequencies as well as (c) percent change of predicted target values with GFP versus native codons, were analysed. Data was subset at the 10th percentile of the measured properties (b, c), which (d) showed very good correlation with predictions on native coding sequences (*n* = 52). Red lines in (a, d) denote least squares fit, TPM transcripts per million.

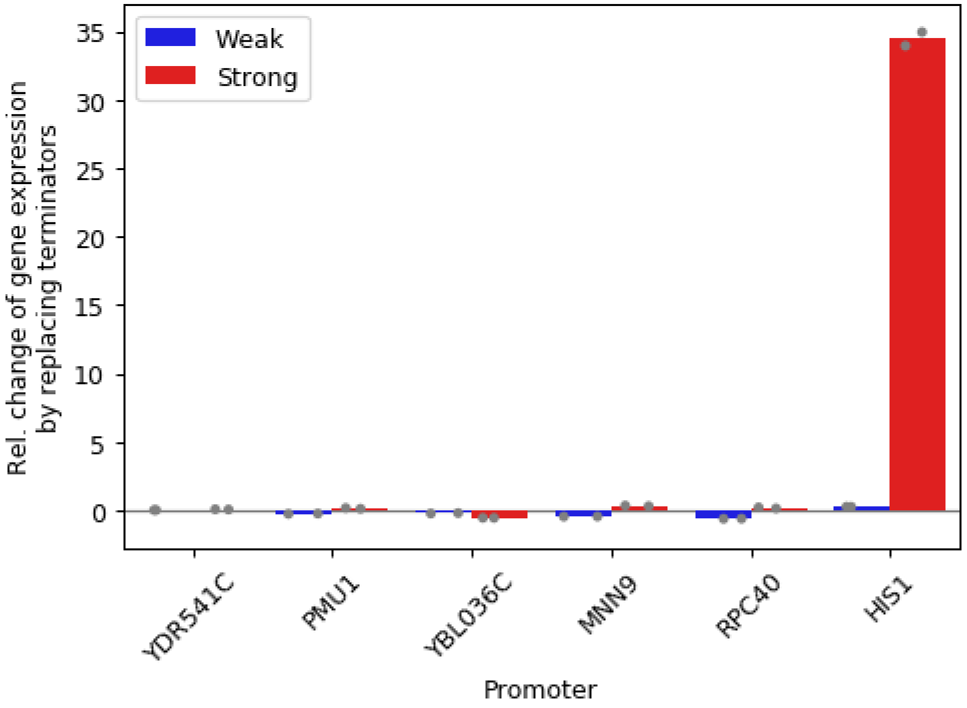

Supplementary figure 33. Relative change in measured GFP fluorescence levels of the constructs, where native terminators were replaced with weak (blue) and strong (red) variants (n = 2 replicates per construct are indicated as grey points, see Results text and Supplementary table 13).

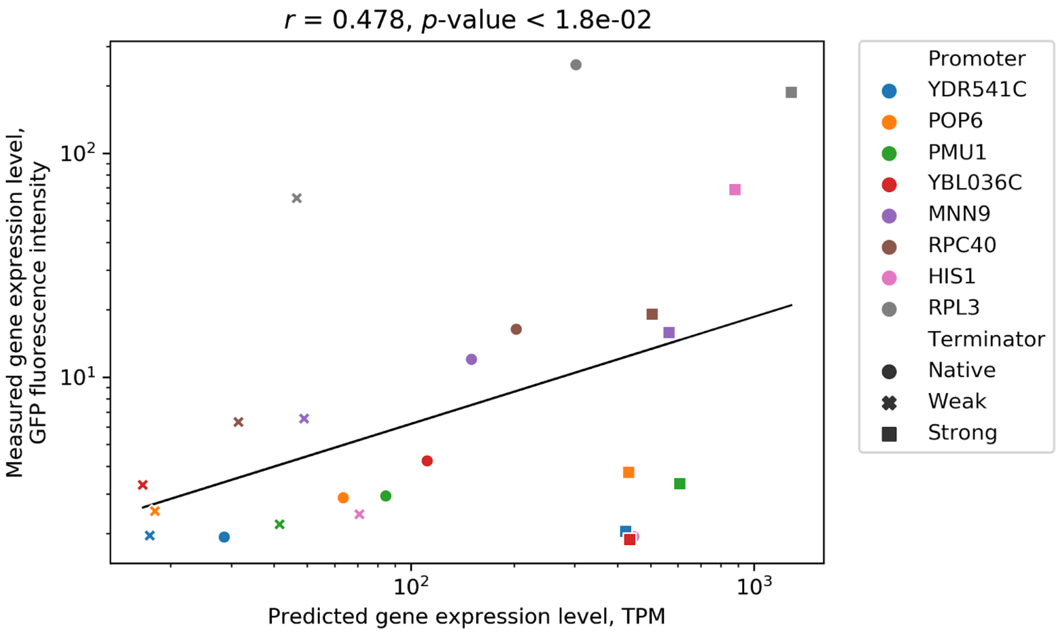

Supplementary figure 34. Experimentally measured GFP fluorescence intensities versus predicted gene expression levels (transcripts per million, TPM) with all tested constructs (n = 24), where constructs of promoters RPL3 and POP6 with more strongly diverging GFP levels than 10th percentile (see Supplementary figure 32) are added. Black line denotes least squares fit. The different promoters are indicated by colors and the different terminators by shapes specified in the figure legend.

a.

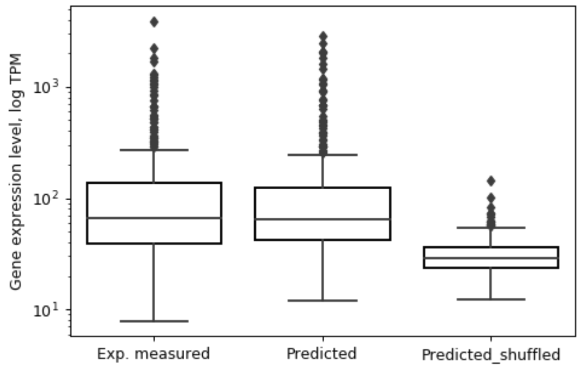

b.

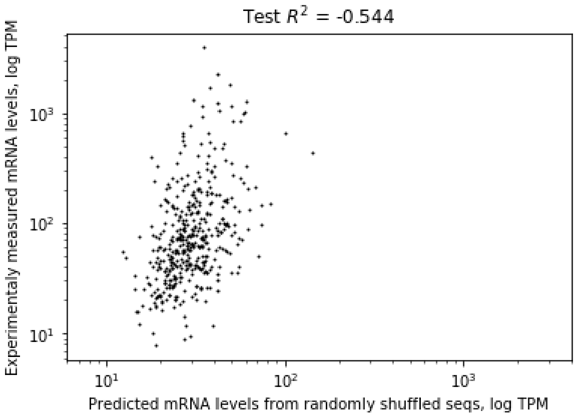

c.

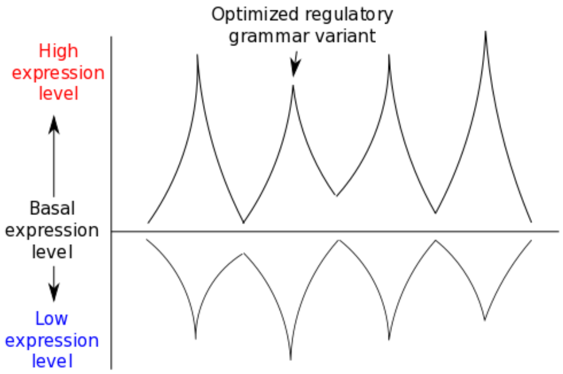

Supplementary figure 35. Prediction of a basal level of *S. cerevisiae* gene expression and a hypothesized regulatory grammar fitness landscape. (a) Predicted expression levels (transcripts per million, TPM) and (b) coefficient of variation R^2 with randomly shuffled sequences with conserved dinucleotide content, compared to non-randomized sequences and the experimentally measured expression levels (n = 425). Median predicted expression level with shuffled sequences was over 2-fold lower than with original ones (64.5 TPM) and suggests that a certain basal level of expression exists that is lower than the organism average but still above zero. Boxes denote interquartile ranges (IQR), centres mark medians and whiskers extend to 1.5 IQR from the quartiles. (c) Hypothesis of a potential evolutionary fitness landscape [39] of regulatory grammar that can be inferred from (a). Grammar optimized for increased expression represents peaks in the landscape, whereas the one with basal lower levels of expression is represented by the valleys. The exception is possibly with very low expression, which again has a more defined grammar, so represented by an inverted valley-to-peak landscape. Whereas both very low and higher expression levels require specially evolved grammar, it can be expected that for the basal expression level regulation is less specific, possibly either 'turned off' or comprising a more diverse grammar.

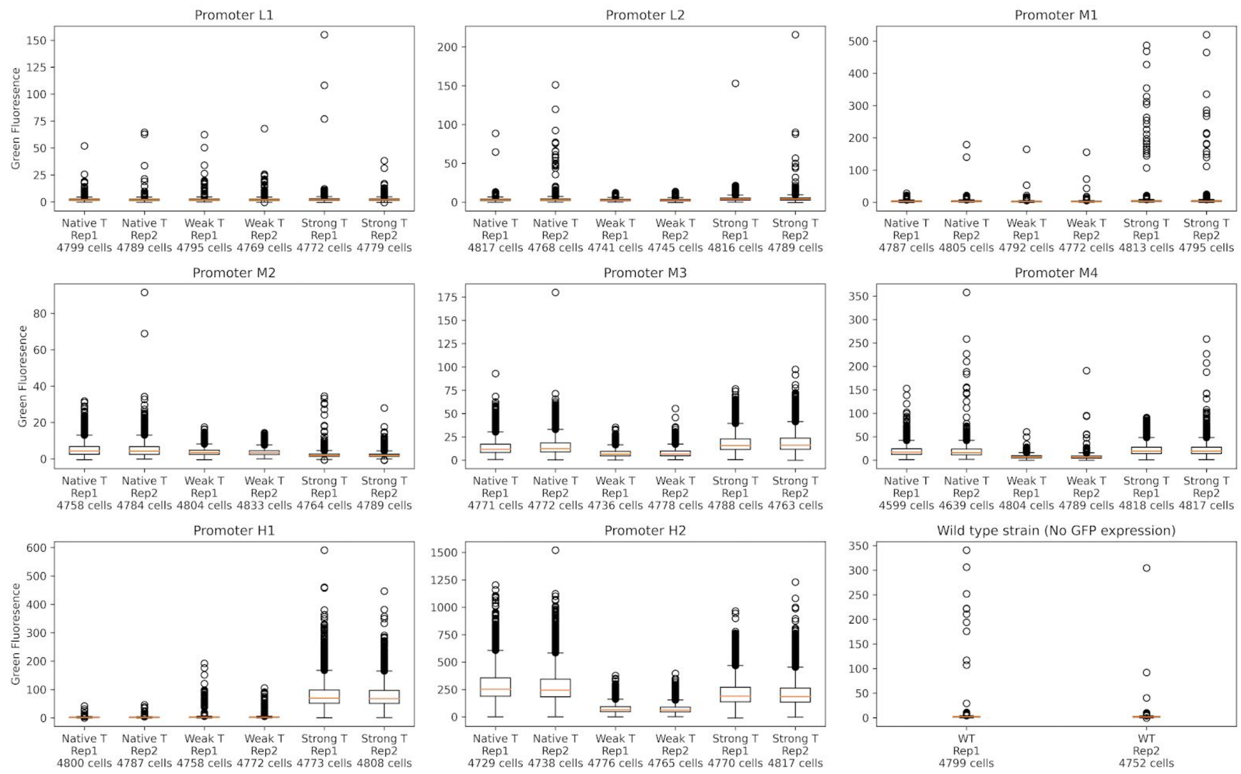

Supplementary figure 36. Distribution of measured GFP fluorescence values for each sample. Samples were grouped together by their used promoter and for each promoter-terminator (native terminator, weak terminator, strong terminator) combination both technical replicates are shown. The number of cells that passed the Forward Scatter / Side Scatter based gate are shown for each sample, 5000 were measured for each.

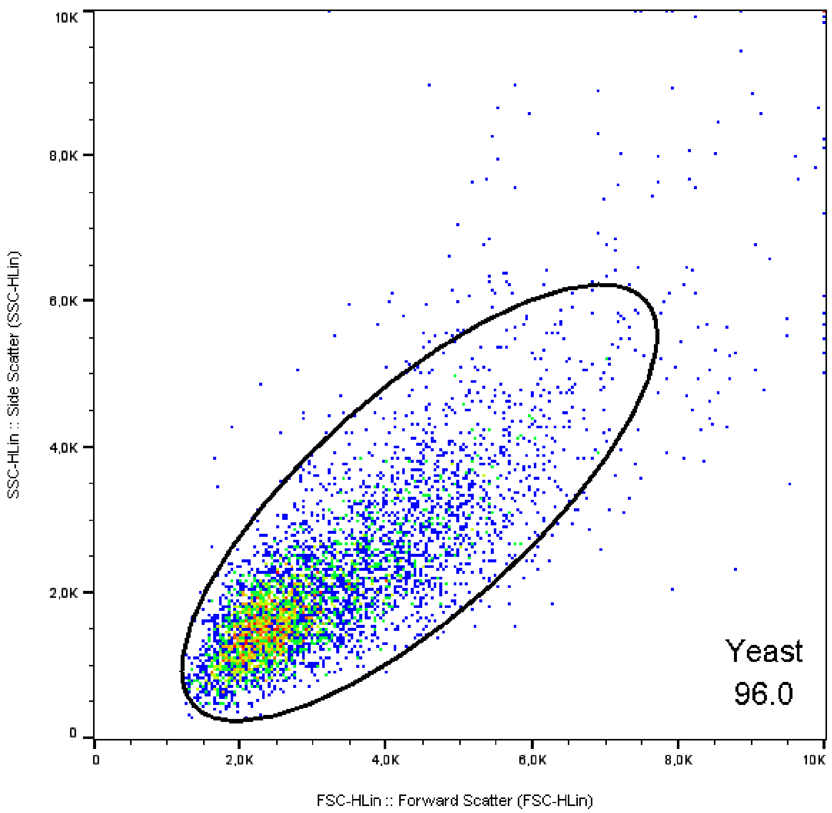

Supplementary figure 37. Overview of the gating strategy used in the flow cytometry experiment. A single gate based on the Forward Scatter and Side Scatter was used to select for yeast cells with a typical yeast cell size and morphology. Across all samples 95.5% of all cells were within this gate.

Supplementary tables

Supplementary table 1. Overview of data and genomic features across the model organisms.

Organism	Common name	Num. coding genes	Genome size (bps)	Coding gene density	Num. RNAseq datasets used	Num. genes with all regions available
E. coli	Bacteria	4,140	4,641,652	892	355	2,665
S. cerevisiae	Yeast	6,600	12,157,105	543	3,025	5,112
A. thaliana	Plant	27,655	135,670,229	204	5,602	22,569
D. melanogaster	Fruitfly	13,931	142,573,024	98	4,410	13,317
D. rerio	Fish	25,592	1,674,207,132	15	1,084	17,526
M. musculus	Mouse	22,604	3,486,944,526	6	2,365	20,244
H. sapiens	Human	20,465	3,609,003,417	6	4,282	18,016
Total	/	120,987	/	/	21,123	99,449
Average	/	17,284	1,295,028,155	252	3,018	14,207

Supplementary table 2. Overview of RNA-seq data across the model organisms.

**Organism**	**Num. active genes *TPM_median* > 5**	**Num. genes *RSD* < 3**	**Num. genes *RSD* < 2**	**Num. genes *RSD* < 1**
E. coli (K12)	2,154	2,012	1,737	932
S. cerevisiae	4,975	4,917	4,804	4,238
A. thaliana	13,814	13,737	13,510	11,719
D. rerio	7,173	7,050	6,719	4,686
D. melanogaster	9,772	9,643	9,227	5,297
M. musculus	9,951	9,785	9,370	6,585
H. sapiens	9,437	9,308	8,893	6,279
Total	57,276	56,452	54,260	39,736
Average	8,182	8,065	7,751	5,677
Relative all	0.644	0.979	0.947	0.665
Relative Prokarya	0.808	0.934	0.806	0.433
Relative Yeast	0.973	0.988	0.966	0.852
Relative Eukarya	0.616	0.987	0.951	0.704

Supplementary table 3. Results of deep modeling across the model organisms. The *p*-values of a two-tailed *F*-test on the test dataset are given for each model.

Organism	*RSD* cutoff	Box-Cox lambda	Train R^2	Validation R^2	Test R^2	Test *p*-value	Test *MSE**
E. coli	*2*	-0.147	0.778	0.645	0.695	< 1e-16	0.170
S. cerevisiae	*1*	0.220	0.841	0.87	0.822	< 1e-16	1.614
A. thaliana	*1*	0.200	0.532	0.424	0.445	< 1e-16	2.835
D. rerio	*1*	0.220	0.771	0.709	0.725	< 1e-16	2.415
D. melanogaster	*1*	0.270	0.753	0.699	0.69	< 1e-16	5.015
M. musculus	*1*	0.120	0.408	0.44	0.394	< 1e-16	1.336
H. sapiens	*1*	0.220	0.466	0.418	0.418	< 1e-16	3.684
Average	/	/	0.650	0.601	0.598	/	2.439

* Mean squared error

Supplementary table 4. Overview of the genomic data resources. Relases 41 and 94 of Ensemble (*S. cerevisiae* and *A. thaliana*) and Ensembl Genomes, respectively, were used. Filenames for each organism correspond to {organism}.{assembly}.{release}.dna.toplevel.fa.gz for genome sequences and {organism}.{assembly}.{release}.gff3.gz for ORFs with Ensemble data.

Organism	Strain	Assembly	Description	Link
Escherichia coli	K-12 MG1655	GCA_000005845.2	Genome sequence	http://regulondb.ccg.unam.mx/menu/download/datasets/files/E_coli_K12_MG1655_U00096.3.txt
			ORF	http://regulondb.ccg.unam.mx/menu/download/datasets/files/Gene_sequence.txt
			UTR	http://regulondb.ccg.unam.mx/menu/download/datasets/files/UTR_5_3_sequence.txt
			Operons	http://regulondb.ccg.unam.mx/menu/download/datasets/files/OperonSet.txt
Saccharomyces cerevisiae	S288C	R64-1-1	ORFs, UTRs	ftp://ftp.ensemblgenomes.org/pub/fungi/release-41/gff3/saccharomyces_cerevisiae
			Genome sequence	ftp://ftp.ensemblgenomes.org/pub/fungi/release-41/fasta/saccharomyces_cerevisiae/dna/
			Additional Xu et al. 2009 UTRs	https://downloads.yeastgenome.org/published_datasets/Xu_2009_PMID_19169243/track_files/Xu_2009_ORF-Ts_V64.gff3
			Additional Nagalakshmi et al. 2008 UTRs	https://science.sciencemag.org/highwire/filestream/589738/field_highwire_adjunct_files/1/1158441_tables_s2_to_s6.zip
Arabidopsis thaliana		TAIR10	ORFs, UTRs	ftp://ftp.ensemblgenomes.org/pub/plants/release-41/gff3/arabidopsis_thaliana
			Genome sequence	ftp://ftp.ensemblgenomes.org/pub/fungi/release-41/fasta/saccharomyces_cerevisiae/dna/
Danio rerio		GRCz11	ORFs, UTRs	ftp://ftp.ensembl.org/pub/release-94/gff3/danio_rerio
			Genome sequence	ftp://ftp.ensembl.org/pub/release-94/fasta/danio_rerio/dna/
Drosophila melanogaster		BDGP6	ORFs, UTRs	ftp://ftp.ensembl.org/pub/release-94/gff3/drosophila_melanogaster
			Genome sequence	ftp://ftp.ensembl.org/pub/release-94/fasta/drosophila_melanogaster/dna/
Mus musculus		GRCm38	ORFs, UTRs	ftp://ftp.ensembl.org/pub/release-94/gff3/mus_musculus

			Genome sequence	ftp://ftp.ensembl.org/pub/release-94/fasta/mus_musculus/dna/
Homo sapiens		GRCh38	ORFs, UTRs	ftp://ftp.ensembl.org/pub/release-94/gff3/homo_sapiens
			Genome sequence	ftp://ftp.ensembl.org/pub/release-94/fasta/homo_sapiens/dna/

Supplementary table 5. Correlations between mRNA stability variables.

Variable 1	Variable 2	Pearson's *r*	*p*-value	R^2
len_3u	gc_3u	0.240	1.00E-16	0.058
gc_c1	gc_c3	0.180	1.00E-16	0.033
len_5u	gc_c2	0.146	1.00E-16	0.021
gc_5u	gc_c3	0.143	1.00E-16	0.020
len_5u	len_cd	0.119	7.26E-15	0.014
gc_3u	gc_c3	0.109	9.50E-13	0.012
len_5u	gc_5u	0.078	4.11E-07	0.006
gc_c2	gc_c3	0.073	2.30E-06	0.005
gc_c1	gc_c2	0.067	1.44E-05	0.004
gc_3u	gc_c1	0.059	1.36E-04	0.003
len_3u	gc_c2	0.042	6.72E-03	0.002
gc_5u	gc_3u	0.041	7.65E-03	0.002
len_cd	gc_5u	0.037	1.57E-02	0.001
gc_5u	gc_c1	0.027	8.39E-02	0.001
len_5u	len_3u	0.012	4.42E-01	0.000
gc_5u	gc_c2	-0.009	5.59E-01	0.000
gc_3u	gc_c2	-0.016	3.09E-01	0.000
len_5u	gc_3u	-0.018	2.52E-01	0.000
len_3u	gc_c1	-0.021	1.71E-01	0.000
len_3u	gc_c3	-0.033	3.19E-02	0.001
len_3u	gc_5u	-0.041	6.93E-03	0.002
len_5u	gc_c3	-0.041	6.92E-03	0.002
len_cd	gc_c2	-0.051	8.09E-04	0.003
len_cd	gc_3u	-0.052	7.74E-04	0.003
len_5u	gc_c1	-0.070	4.37E-06	0.005
len_cd	len_3u	-0.079	2.29E-07	0.006
len_cd	gc_c1	-0.163	1.00E-16	0.027
len_cd	gc_c3	-0.297	1.00E-16	0.088

Supplementary table 6. Hyper-parameters used with deep learning algorithms. CNN denotes convolutional neural networks, RNN recurrent neural networks and FC fully connected neural networks.

Type	Parameter name	Values	Value range
Global	num epochs	500	fixed
	early stopping min delta	0.01	fixed
	early stopping patience	50	fixed
	LRS* epoch drop	10	fixed
	learning rate	(0.00001,0.1)	log variable
	beta_1	(0.5,0.95)	uniform variable
	beta_2	(0.9,0.95)	uniform variable
	epsilon	1.00E-07	fixed
	mbatch	[64,128, 256]	fixed
CNN	kernel size	[10, 20, 30, 40]	fixed
	filters	[32, 64, 128]	fixed
	dilation	[1, 2, 4]	fixed
	stride	1	fixed
	max-pool size	[1, 2, 4]	fixed
	max-pool stride	[1, 2]	fixed
	dropout	(0, 1)	uniform variable
RNN	kernel size	64	fixed
	dropout	(0, 1)	uniform variable
FC	dense size	[32, 64, 128]	fixed
	dropout	(0, 1)	uniform variable

* Learning rate scheduler

Supplementary table 7. Additional data used in the study.

Description	Link
Yeast TFIID/SAGA promoters [34]	https://ars.els-cdn.com/content/image/1-s2.0-S1097276504000875-mmc2.xls
SGD GO slim terms [40]	http://sgd-archive.yeastgenome.org/curation/literature/go_slim_mapping.tab
Yeast exp. fluorescence measurements with varying promoters [19]	https://www.embopress.org/action/downloadSupplement?doi=10.1038%2Fmsb.2013.59&file=msb201359-sup-0002.xlsx
Yeast exp. fluorescence measurements with varying terminators [20]	https://ndownloader.figstatic.com/files/4043311
Yeast exp. fluorescence measurements with de novo sequences [41]	https://github.com/Carldeboer/CisRegModels/blob/master/example/HighQuality.pTpA.Glu.test.txt.gz
Yeast nucleosome occupancy scores [33]	https://static-content.springer.com/esm/art%3A10.1038%2Fsrep33970/MediaObjects/41598_2016_BFsrep33970_MOESM3_ESM.xls
Yeast OPN/DPN regulation strategy [42]	https://genome.cshlp.org/content/suppl/2008/08/08/gr.076059.108.DC1/Supplementary_Figures_april15.pdf
Yeast Jaspar DNA seq motifs (JASPAR2018_CORE_fungi_non-redundant.meme) [35]	http://meme-suite.org/meme-software/Databases/motifs/motif_databases.12.19.tgz
Yeastract DNA seq motifs (YEASTRACT_20130918.meme) [36]	http://meme-suite.org/meme-software/Databases/motifs/motif_databases.12.19.tgz
SGD gene names [40]	https://downloads.yeastgenome.org/curation/chromosomal_feature/SGD_features.tab
SGD motif information [40]	https://www.yeastgenome.org
Fungi protein orthologs data [43]	ftp://ftp.ensemblgenomes.org/pub/fungi/release-41/tsv/ensembl-compara/homologies/Compara.94.protein_default.homologies.tsv.gz
Transcriptomics data all organisms [44]	http://dee2.io/mx/

Supplementary table 8. Deep modeling results using different combinations of codon probabilities, mRNA stability variables and regulatory sequences. The *p*-values of a two-tailed *F*-test on the test dataset are given for each model.

**Input variable combinations**	**Target**	**Layer type**	**Input type**	**Train R^2**	**Validation R^2**	**Test R^2**	**Test *p*-value**	**Test *MSE****
Regulatory regions	TPM	CNN	Sequences	0.845	0.575	0.492	< 1e-16	4.609
mRNA stability	TPM	Dense (FC)	8 variables	0.386	0.471	0.378	< 1e-16	5.641
Coding regions	TPM	Dense (FC)	64 variables	0.715	0.742	0.69	< 1e-16	0.037
Regulatory + stability	TPM	Dense (FC)	72 variables	0.597	0.603	0.558	< 1e-16	4.004
Regulatory + coding	TPM	CNN + Dense	Seq. + 64 vars.	0.824	0.862	0.816	< 1e-16	1.669
Codoning + stability	TPM	Dense (FC)	72 variables	0.721	0.751	0.755	< 1e-16	0.030
All	TPM	CNN + Dense	Seq. + 72 vars.	0.841	0.87	0.822	< 1e-16	1.614
Regulatory regions	Codon prob.	CNN + Dense	Sequences	0.538	0.543	0.582	< 1e-16	0.000
Regulatory regions	mRNA stability vars.	CNN + Dense	Sequences	0.969	0.776	0.779	< 1e-16	0.003

* Mean squared error

Supplementary table 9. Shallow modeling results using linear regression with different combinations of codon probabilities, mRNA stability variables and kmers of size 4 to 6 as features.

Features	Kmer size	Train R^2	Test R^2	Train *MSE**	Test *MSE*	Fit time	Score time
codon_stability	4	0.699	0.685	0.039	0.040	0.030	0.002
codon	4	0.693	0.681	0.039	0.041	0.037	0.002
codon_stability_kmers	4	0.728	0.674	0.035	0.042	0.456	0.005
codon_kmers	4	0.720	0.667	0.036	0.043	0.470	0.007
stability_kmers	4	0.265	0.159	0.094	0.108	0.325	0.005
stability	4	0.147	0.142	0.109	0.110	0.002	0.001
kmers	4	0.153	0.031	0.109	0.124	0.409	0.005
codon_stability	5	0.699	0.685	0.039	0.040	0.077	0.002
codon	5	0.693	0.681	0.039	0.041	0.018	0.002
codon_stability_kmers	5	0.792	0.593	0.027	0.052	7.497	0.018
codon_kmers	5	0.788	0.585	0.027	0.053	6.992	0.016
stability	5	0.147	0.142	0.109	0.110	0.002	0.001
stability_kmers	5	0.423	-0.085	0.074	0.139	5.278	0.015
kmers	5	0.343	-0.234	0.084	0.158	6.558	0.018
codon_stability	6	0.699	0.685	0.039	0.040	0.057	0.002
codon	6	0.693	0.681	0.039	0.041	0.021	0.002
stability	6	0.147	0.142	0.109	0.110	0.002	0.001
codon_stability_kmers	6	1.000	-8.008	0.000	1.150	234.612	0.060
codon_kmers	6	1.000	-8.313	0.000	1.188	237.973	0.065
stability_kmers	6	1.000	-15.425	0.000	2.097	230.862	0.056
kmers	6	1.000	-17.296	0.000	2.333	235.376	0.068

* Mean squared error

Supplementary table 10. 14 yeast species used to analyse co-evolution of regulatory and coding regions. Release 41 of Ensemble was used. Filenames for each organism correspond to {organism}.{assembly}.{release}.dna.toplevel.fa.gz for genome sequences and {organism}.{assembly}.{release}.gff3.gz for ORFs.

Clade [45]	Species	Strain	Assembly	Genome sequence link	ORFs link
Saccharomyces	Saccharomyces cerevisiae	S288C	R64-1-1	ftp://ftp.ensemblgenomes.org/pub/fungi/release-41/fasta/saccharomyces_cerevisiae/dna/	ftp://ftp.ensemblgenomes.org/pub/fungi/release-41/gff3/saccharomyces_cerevisiae
Saccharomyces	Saccharomyces eubayanus	FM1318	SEUB3.0	ftp://ftp.ensemblgenomes.org/pub/fungi/release-41/fasta/fungi_ascomycota3_collection/saccharomyces_eubayanus_gca_001298625/dna/	ftp://ftp.ensemblgenomes.org/pub/fungi/release-41/gff3/fungi_ascomycota3_collection/saccharomyces_eubayanus_gca_001298625
	Candida glabrata	CSB 138	ASM254v2	ftp://ftp.ensemblgenomes.org/pub/fungi/release-41/fasta/fungi_ascomycota1_collection/_candida_glabrata_gca_000002545/dna/	ftp://ftp.ensemblgenomes.org/pub/fungi/release-41/gff3/fungi_ascomycota1_collection/_candida_glabrata_gca_000002545
Kluyveromyces	Kluyveromyces lactis	NRRL Y-1140	ASM251v1	ftp://ftp.ensemblgenomes.org/pub/fungi/release-41/fasta/fungi_ascomycota1_collection/kluyveromyces_lactis_gca_000002515/dna/	ftp://ftp.ensemblgenomes.org/pub/fungi/release-41/gff3/fungi_ascomycota1_collection/kluyveromyces_lactis_gca_000002515
Candida	Candida albicans	SC 5314	Cand_albi_SC5314_V4	ftp://ftp.ensemblgenomes.org/pub/fungi/release-41/fasta/fungi_ascomycota2_collection/candida_albicans_sc5314_gca_000784635/dna/	ftp://ftp.ensemblgenomes.org/pub/fungi/release-41/gff3/fungi_ascomycota2_collection/candida_albicans_sc5314_gca_000784635
Candida	Debaryomyces hansenii	CBS767	ASM644v2	ftp://ftp.ensemblgenomes.org/pub/fungi/release-41/fasta/fungi_ascomycota1_collection/debaryomyces_hansenii_cbs767_gca_000006445/dna/	ftp://ftp.ensemblgenomes.org/pub/fungi/release-41/gff3/fungi_ascomycota1_collection/debaryomyces_hansenii_cbs767_gca_000006445
	Yarrowia lipolytica		YALIA101	ftp://ftp.ensemblgenomes.org/pub/fungi/release-41/fasta/fungi_ascomycota3_collection/yarrowia_lipolytica_gca_900087985/dna/	ftp://ftp.ensemblgenomes.org/pub/fungi/release-41/gff3/fungi_ascomycota3_collection/yarrowia_lipolytica_gca_900087985
Schizosaccharomyces	Schizosaccharomyces pombe	972h-	ASM294v2	ftp://ftp.ensemblgenomes.org/pub/fungi/release-41/fast	ftp://ftp.ensemblgenomes.org/pub/fungi/release-41

				a/schizosaccharomyces_pombe/dna/	/gff3/schizosaccharomyces_pombe
Schizosaccharomyces	Schizosaccharomyces japonicus	YFS 275	GCA_000149845.2	ftp://ftp.ensemblgenomes.org/pub/fungi/release-41/fasta/schizosaccharomyces_japonicus/dna/	ftp://ftp.ensemblgenomes.org/pub/fungi/release-41/gff3/schizosaccharomyces_japonicus
	Saccharomyces kudriavzevii	IFO 1802	Saccharomyces_kudriavzevii_strain_IFO1802_v1.0	ftp://ftp.ensemblgenomes.org/pub/fungi/release-41/fasta/fungi_ascomycota1_collection/saccharomyces_kudriavzevii_ifo_1802_gca_000167075/dna/	ftp://ftp.ensemblgenomes.org/pub/fungi/release-41/gff3/fungi_ascomycota1_collection/saccharomyces_kudriavzevii_ifo_1802_gca_000167075
	Saccharomyces arboricola	H-6	SacArb1.0	ftp://ftp.ensemblgenomes.org/pub/fungi/release-41/fasta/fungi_ascomycota1_collection/saccharomyces_arboricola_h_6_gca_000292725/dna/	ftp://ftp.ensemblgenomes.org/pub/fungi/release-41/gff3/fungi_ascomycota1_collection/saccharomyces_arboricola_h_6_gca_000292725
	Saccharomyces sp boulardii	biocodex	ASM129837v2	ftp://ftp.ensemblgenomes.org/pub/fungi/release-41/fasta/fungi_ascomycota3_collection/saccharomyces_sp_boulardii__gca_001298375/dna/	ftp://ftp.ensemblgenomes.org/pub/fungi/release-41/gff3/fungi_ascomycota3_collection/saccharomyces_sp_boulardii__gca_001298375
	Kluyveromyces marxianus	DMKU3 1042	Kmar_1.0	ftp://ftp.ensemblgenomes.org/pub/fungi/release-41/fasta/fungi_ascomycota3_collection/kluyveromyces_marxianus_dmku3_1042_gca_001417885/dna/	ftp://ftp.ensemblgenomes.org/pub/fungi/release-41/gff3/fungi_ascomycota3_collection/kluyveromyces_marxianus_dmku3_1042_gca_001417885
	Kluyveromyces _dobzhanskii	CBS 2104	KLDO_01	ftp://ftp.ensemblgenomes.org/pub/fungi/release-41/fasta/fungi_ascomycota3_collection/kluyveromyces_dobzhanskii_cbs_2104_gca_000820885/dna/	ftp://ftp.ensemblgenomes.org/pub/fungi/release-41/gff3/fungi_ascomycota3_collection/kluyveromyces_dobzhanskii_cbs_2104_gca_000820885

Supplementary table 11. Construction of regulatory DNA motifs at different sequence identity cutoffs.

Seq. id.	Num. motifs	% Relevant sequences in motifs	% Jaspar targets	% Motif overlap between gene regions	Num. co-occurring motifs
0.8	2,210	0.440	0.318	0.153	116,734
0.85	2,786	0.272	0.284	0.269	12,809
0.9	1,152	0.082	0.210	0.140	408

Supplementary table 12. Groups of motif co-occurrence rules with a common Jaspar TFBS motif in promoter regions that define expression levels in an over 30 fold range of values.

Motif name	BH adj. *p*-value	Regions with differing motifs	Num. rules	Num. genes	Fold change
NHP6B	4.49E-03	(3UTR, 5UTR, Promoter, Terminator)	144	144	648.016
ABF1	5.00E-02	(3UTR, 5UTR, Promoter, Terminator)	32	42	298.673
STB3	6.62E-04	(3UTR, 5UTR, Promoter, Terminator)	46	64	166.031
HAP3	3.59E-02	(3UTR, 5UTR, Promoter, Terminator)	83	102	132.435
AZF1	3.34E-02	(3UTR, 5UTR, Promoter, Terminator)	5	12	100.093
CBF1	3.70E-03	(3UTR, 5UTR, Promoter, Terminator)	58	65	73.371
CUP2	9.76E-04	(3UTR, 5UTR, Promoter, Terminator)	3	21	55.298
CUP9	2.29E-02	(3UTR, 5UTR, Promoter, Terminator)	54	77	53.290
SFP1	2.01E-03	(3UTR, 5UTR, Promoter, Terminator)	7	14	51.683
RSC3	4.23E-02	(3UTR, 5UTR, Promoter, Terminator)	10	17	42.286
SUM1	3.85E-02	(3UTR, 5UTR, Promoter, Terminator)	10	15	35.595
NSI1	1.78E-02	(3UTR, 5UTR, Promoter, Terminator)	18	29	34.999

Supplementary table 13. Experimentally tested gene regulatory structure constructs.

Dataset	Gene ID	Standard gene name	Native *TPM*	Native *TPM* with gfp	Euclidean dist. to GFP	Fold change with GFP	*TPM* X (term)	*TPM* Y (term)	Fold change X	Fold change Y
GFP codon properties within 10% of native ones	YDR541C	YDR541C	26.43	28.65	24.80	0.03	17.39	422.84	0.61	14.76
	YKL128C	PMU1	75.83	84.67	26.80	0.04	41.52	608.29	0.49	7.18
	YBL036C	YBL036C	107.74	111.75	27.64	0.01	16.58	433.95	0.15	3.88
	YPL050C	MNN9	147.64	150.40	29.60	0.01	48.95	564.83	0.33	3.76
	YPR110C	RPC40	208.47	203.14	29.80	0.01	31.52	504.77	0.16	2.48
	YER055C	HIS1	420.27	447.07	25.92	0.02	70.93	880.93	0.16	1.97
Weak prom	YGR030C	POP6	27.18	63.63	35.37	1.93	18.00	430.94	0.28	6.77
Strong prom	YOR063W	RPL3	3,886.98	303.20	61.55	12.03	46.60	1,284.72	0.15	4.24
Weak term (X)	YPR153W	MAY24	8.78	11.66	41.12	0.17	/	/	/	/
Strong term (Y)	YLR167W	RPS31	5,823.21	4,511.36	41.36	0.06	/	/	/	/

Supplementary table 14. List of PCR primers.

Primer	Sequence
promoter_YPR153W_fwd	TAGGCAAAAGCCAAGGAGCGTTTGCCATGAACTTCCACAATCGTTGTA TATTATTAAGTGCCAA
promoter_YPR153W_rev	TTATGGTTTTACCGGTCAAAGTCTTGACGAAAATCTGCATTAATACGGC AGGAAGTTGGA
promoter_YGR030C_fwd	TAGGCAAAAGCCAAGGAGCGTTTGCCATGAACTTCCACAATCTCTTGA TTATGTCATATGAAAGG
promoter_YGR030C_rev	TTATGGTTTTACCGGTCAAAGTCTTGACGAAAATCTGCATTTTTGATTT GCTTTTATCTTTTTTTCT
promoter_YLR167W_fwd	TAGGCAAAAGCCAAGGAGCGTTTGCCATGAACTTCCACAAAGTAAGTA AAAACATTTGAGCCTC
promoter_YLR167W_rev	TTATGGTTTTACCGGTCAAAGTCTTGACGAAAATCTGCATTCTTGGCTT GTCGGCAAA
promoter_YBL036C_fwd	TAGGCAAAAGCCAAGGAGCGTTTGCCATGAACTTCCACAATAACAGGG GATCCTATGCA
promoter_YBL036C_rev	TTATGGTTTTACCGGTCAAAGTCTTGACGAAAATCTGCATTATTGCAAT GTGAATGCTGG
promoter_YPL050C_fwd	TAGGCAAAAGCCAAGGAGCGTTTGCCATGAACTTCCACAAAGACAAGA AAATGTTTATGAGCAT
promoter_YPL050C_rev	TTATGGTTTTACCGGTCAAAGTCTTGACGAAAATCTGCATTTGTTTCCT AACTTTTTATTCTAGC
promoter_YER055C_fwd	TAGGCAAAAGCCAAGGAGCGTTTGCCATGAACTTCCACAACTAATTGA GACTTTGTGGCC
promoter_YER055C_rev	TTATGGTTTTACCGGTCAAAGTCTTGACGAAAATCTGCATTTTTTCTATT GAATTTTTTAGAAACC
promoter_YPR110C_fwd	TAGGCAAAAGCCAAGGAGCGTTTGCCATGAACTTCCACAAGGTTGCTA ATCACTATTGGAG
promoter_YPR110C_rev	TTATGGTTTTACCGGTCAAAGTCTTGACGAAAATCTGCATTATCTTCTT TCACCTACTTACTTT
promoter_YDR541C_fwd	TAGGCAAAAGCCAAGGAGCGTTTGCCATGAACTTCCACAATTTAAGAC TCTAGAGCCAACG
promoter_YDR541C_rev	TTATGGTTTTACCGGTCAAAGTCTTGACGAAAATCTGCATTGGTGTGAA ACGAACGAAA
promoter_YKL128C_fwd	TAGGCAAAAGCCAAGGAGCGTTTGCCATGAACTTCCACAATTAATACT GCTACCATTTCTTCC
promoter_YKL128C_rev	TTATGGTTTTACCGGTCAAAGTCTTGACGAAAATCTGCATTTGTTGAAT AACTGTTGGTGA
promoter_YOR063W_fwd	TAGGCAAAAGCCAAGGAGCGTTTGCCATGAACTTCCACAATTATTAAA TTCAGTGGTAATGCAA
promoter_YOR063W_rev	TTATGGTTTTACCGGTCAAAGTCTTGACGAAAATCTGCATTGATTGATT GTTGTAGTAACTGTG
terminator_YPR153W_fwd	TGCTGGGATTACACATGGCATGGATGAACTATACAAATAGTACTGATTT GATGATAAAAGTTAGC

terminator_YPR153W_rev	ACATCTAAACTTTTTAATATCTGAAAGCGCTAGTCGTGTGCCATCTTGTTGAGGATCAAA
terminator_YGR030C_fwd	TGCTGGGATTACACATGGCATGGATGAACTATACAAATAGAATCGACCAGCTCTTTTAGCA
terminator_YGR030C_rev	ACATCTAAACTTTTTAATATCTGAAAGCGCTAGTCGTGTGGGATTCAAAGCGAGGCCTA
terminator_YLR167W_fwd	TGCTGGGATTACACATGGCATGGATGAACTATACAAATAGAGTAAAGTATTTTTAAAACTTATATATTTT
terminator_YLR167W_rev	ACATCTAAACTTTTTAATATCTGAAAGCGCTAGTCGTGTGAACGCTAAAAAGGGTAAAAT
terminator_YBL036C_fwd	TGCTGGGATTACACATGGCATGGATGAACTATACAAATAGGTAGGTTGAATGAACTGAGATTTT
terminator_YBL036C_rev	ACATCTAAACTTTTTAATATCTGAAAGCGCTAGTCGTGTGGGGCTTTGATATAGTCGATC
terminator_YPL050C_fwd	TGCTGGGATTACACATGGCATGGATGAACTATACAAATAGAGCAACTGAGCAAAAAGCA
terminator_YPL050C_rev	ACATCTAAACTTTTTAATATCTGAAAGCGCTAGTCGTGTGGATAGAATGGAAGTACAAGATATAAA
terminator_YER055C_fwd	TGCTGGGATTACACATGGCATGGATGAACTATACAAATAGAGATAGAACAGAAAAAGGGAAG
terminator_YER055C_rev	ACATCTAAACTTTTTAATATCTGAAAGCGCTAGTCGTGTGACAGCTTTATGCGTTACGAT
terminator_YPR110C_fwd	TGCTGGGATTACACATGGCATGGATGAACTATACAAATAGATCCTACTTTGCATACTAATAAAA
terminator_YPR110C_rev	ACATCTAAACTTTTTAATATCTGAAAGCGCTAGTCGTGTGTTTACTTTATTTTCACTAACATGTG
terminator_YDR541C_fwd	TGCTGGGATTACACATGGCATGGATGAACTATACAAATAGACGCCATACCACACATAATC
terminator_YDR541C_rev	ACATCTAAACTTTTTAATATCTGAAAGCGCTAGTCGTGTGCCAAATTATCCCTGTACTCTTG
terminator_YKL128C_fwd	TGCTGGGATTACACATGGCATGGATGAACTATACAAATAGATGTCCACTCCCTCTTTTATACTA
terminator_YKL128C_rev	ACATCTAAACTTTTTAATATCTGAAAGCGCTAGTCGTGTGTCTTCTTGGGCTCCTTAACG
terminator_YOR063W_fwd	TGCTGGGATTACACATGGCATGGATGAACTATACAAATAGAGAAGTTTTGTTAGAAAATAAATCATTTTT
terminator_YOR063W_rev	ACATCTAAACTTTTTAATATCTGAAAGCGCTAGTCGTGTGGGCTTGTCCCTTCGAGTG

Supplementary table 15. List of used constructs [46].

Construct	Sequence
*UBIMΔkGFP**	atgcagattttcgtcaagactttgaccggtaaaaccataacat tggaagttgaatcttccgataccatcgacaacgttaagtcga aaattcaagacaaggaaggtatccctccagatcaacaaag attgatctttgccggtaagcagctagaagacggtagaacgct gtctgattacaacattcagaaggagtccaccttacatcttgtgc taaggctaagaggtggtatgcacggatccggagcttggctgt tgcccgtctcactggtgaaaagaaaaaccaccctggcgccc aatacgagtaaaggagaagaacttttcactggagttgtccca attcttgttgaattagatggtgatgttaatgggcacaaattttctg tcagtggagagggtgaaggtgatgcaacatacggaaaactt acccttaaatttatttgcactactggaaaactacctgttccatgg ccaacacttgtcactactctcacttatggtgttcaatgcttttcaa gatacccagatcacatgaaacagcatgactttttcaagagtg ccatgcccgaaggttatgtacaggaaagaactatatttttcaa agatgacgggaactacaagacacgtgctgaagtcaagtttg aaggtgatacccttgttaatagaatcgagttaaaaggtattga ttttaaagaagatggaaacattcttggacacaaattggaatac aactataactcacacaatgtatacatcatggcagacaaaca aaagaatggaatcaaagctaacttcaaaattagacacaac attgaagatggaagcgttcaactagcagaccattatcaaca aaatactccaattggcgatggccctgtccttttaccagacaac cattacctgtccacacaatctgccctttcgaaagatcccaacg aaaagagagaccacatggtccttcttgagtttgtaacagctg ctgggattacacatggcatggatgaactatacaaatag

Supplementary table 16. Minimal Media.

	Minimal Media [Recipe for 1 liter]
KH_2PO_4	14.4 g
$MgSO_4$	0.5 g
(NH4)2SO4	7.5 g
Glucose 40%	50 ml
Trace metals stock solution*	1 ml
Vitamin stock solution**	1 ml

*Trace metal stock solution components (per liter of stock solution): 15.0 g EDTA-Na_2, 4.5 g $CaCl_2$·2H2O, 4.5 g $ZnSO_4$·7H2O, 3 g $FeSO_4$·7H2O, 1g H_3BO_3, 0.84 g $MnCl_2$·2H2O, 0.4 g Na_2MoO_4·2H2O, 0.3 g $CuSO_4$·5H2O, 0.3 g $CoCl_2$·6H2O and 0.1 g KI.
**Vitamin stock solution components (per liter of stock solution): 25 g myo-inositol, 1 g nicotinic acid, 1 g calcium pantothenate, 1 g pyridoxine HCl, 1 g thiamine HCl, 0.2 g 4-aminobenzoic acid and 0.05 g biotin. The pH of the media was adjusted to 6.3-6.4 using KOH pellets.

Supplementary table 17. Author contributions as defined by the CRediT taxonomy (https://casrai.org/credit/).

Author	Conceptualization	Data curation	Formal Analysis	Funding acquisition	Investigation	Methodology	Project administration	Resources	Software	Supervision	Validation	Visualization	Writing – original draft	Writing – review & editing
JZ	x	x	x		x	x		x	x		x	x	x	x
CB			x		x	x		x			x			x
FB		x				x			x					x
AMS		x	x		x	x			x					x
RC		x	x		x	x			x					
VS						x								x
VV				x			x			x				
JN				x			x			x				
MT			x	x		x	x		x					x
AZ	x	x	x	x	x	x	x	x		x		x	x	x

Supplementary references

1. Espinar, L., Schikora Tamarit, M. À., Domingo, J. & Carey, L. B. Promoter architecture determines cotranslational regulation of mRNA. *Genome Res.* **28**, 509–518 (2018).
2. Dvir, S., Velten, L., Sharon, E. & Zeevi, D. Deciphering the rules by which 5′-UTR sequences affect protein expression in yeast. *Proc. Natl. Acad. Sci.* **110**, E2792–E2801 (2013).
3. Cuperus, J. T., Groves, B. & Kuchina, A. Deep learning of the regulatory grammar of yeast 5′ untranslated regions from 500,000 random sequences. *Genome Res.* **27**, 1–10 (2017).
4. Cheng, J., Maier, K. C., Avsec, Ž., Rus, P. & Gagneur, J. Cis-regulatory elements explain most of the mRNA stability variation across genes in yeast. *RNA* **23**, 1648–1659 (2017).
5. Shalem, O. *et al.* Systematic dissection of the sequence determinants of gene 3'end mediated expression control. *PLoS Genet.* **11**, e1005147 (2015).
6. Morse, N. J., Gopal, M. R., Wagner, J. M. & Alper, H. S. Yeast Terminator Function Can Be Modulated and Designed on the Basis of Predictions of Nucleosome Occupancy. *ACS Synth. Biol.* **6**, 2086–2095 (2017).
7. Lubliner, S. *et al.* Core promoter sequence in yeast is a major determinant of expression level. *Genome Res.* **25**, 1008–1017 (2015).
8. Sharon, E. *et al.* Inferring gene regulatory logic from high-throughput measurements of thousands of systematically designed promoters. *Nat. Biotechnol.* **30**, 521–530 (2012).
9. Redden, H. & Alper, H. S. The development and characterization of synthetic minimal yeast promoters. *Nat. Commun.* **6**, 7810 (2015).
10. Li, J., Liang, Q., Song, W. & Marchisio, M. A. Nucleotides upstream of the Kozak sequence strongly influence gene expression in the yeast S. cerevisiae. *J. Biol. Eng.* **11**, 25 (2017).
11. Zhou, Z., Dang, Y., Zhou, M., Yuan, H. & Liu, Y. Codon usage biases co-evolve with transcription termination machinery to suppress premature cleavage and polyadenylation. *Elife* **7**, (2018).
12. Watson, J. D. *et al. Molecular Biology of the Gene. 6th. ed*. (Pearson/Benjamin Cummings, 2008).
13. Moqtaderi, Z., Geisberg, J. V., Jin, Y., Fan, X. & Struhl, K. Species-specific factors mediate extensive heterogeneity of mRNA 3′ ends in yeasts. *Proc. Natl. Acad. Sci. U. S. A.* **110**, 11073–11078 (2013).
14. Curran, K. A. *et al.* Short Synthetic Terminators for Improved Heterologous Gene Expression in Yeast. *ACS Synth. Biol.* **4**, 824–832 (2015).
15. Curran, K. A. *et al.* Design of synthetic yeast promoters via tuning of nucleosome architecture. *Nat. Commun.* **5**, 4002 (2014).
16. Neymotin, B., Ettorre, V. & Gresham, D. Multiple Transcript Properties Related to Translation Affect mRNA Degradation Rates in Saccharomyces cerevisiae. *G3* **6**, 3475–3483 (2016).
17. Ashburner, M. *et al.* Gene ontology: tool for the unification of biology. The Gene Ontology Consortium. *Nat. Genet.* **25**, 25–29 (2000).

18. The Gene Ontology Consortium & The Gene Ontology Consortium. The Gene Ontology Resource: 20 years and still GOing strong. *Nucleic Acids Res.* vol. 47 D330–D338 (2019).
19. Keren, L. *et al.* Promoters maintain their relative activity levels under different growth conditions. *Mol. Syst. Biol.* **9**, 701 (2013).
20. Yamanishi, M. *et al.* A Genome-Wide Activity Assessment of Terminator Regions in Saccharomyces cerevisiae Provides a ″Terminatome″ Toolbox. *ACS Synth. Biol.* **2**, 337–347 (2013).
21. de Boer, C. G. *et al.* Deciphering eukaryotic gene-regulatory logic with 100 million random promoters. *Nat. Biotechnol.* **38**, 56–65 (2020).
22. Jenjaroenpun, P. *et al.* Complete genomic and transcriptional landscape analysis using third-generation sequencing: a case study of Saccharomyces cerevisiae CEN. PK113-7D. *Nucleic Acids Res.* **46**, e38–e38 (2018).
23. Koonin, E. V. & Wolf, Y. I. Genomics of bacteria and archaea: the emerging dynamic view of the prokaryotic world. *Nucleic Acids Res.* **36**, 6688–6719 (2008).
24. Lynch, M. & Conery, J. S. The origins of genome complexity. *Science* **302**, 1401–1404 (2003).
25. Pelechano, V., García-Martínez, J. & Pérez-Ortín, J. E. A genomic study of the inter-ORF distances inSaccharomyces cerevisiae. *Yeast* vol. 23 689–699 (2006).
26. Zicola, J., Liu, L., Tänzler, P. & Turck, F. Targeted DNA methylation represses two enhancers of FLOWERING LOCUS T in Arabidopsis thaliana. *Nat Plants* **5**, 300–307 (2019).
27. Clément, Y., Torbey, P. & Gilardi-Hebenstreit, P. Genome-wide enhancer-gene regulatory maps in two vertebrate genomes. *bioRxiv* (2018).
28. Chepelev, I., Wei, G., Wangsa, D., Tang, Q. & Zhao, K. Characterization of genome-wide enhancer-promoter interactions reveals co-expression of interacting genes and modes of higher order chromatin organization. *Cell Research* vol. 22 490–503 (2012).
29. Mora, A., Sandve, G. K., Gabrielsen, O. S. & Eskeland, R. In the loop: promoter-enhancer interactions and bioinformatics. *Brief. Bioinform.* **17**, 980–995 (2016).
30. Zeiler, M. D. & Fergus, R. Visualizing and Understanding Convolutional Networks. in *Computer Vision – ECCV 2014* 818–833 (Springer International Publishing, 2014).
31. Ancona, M., Ceolini, E., Öztireli, C. & Gross, M. Towards better understanding of gradient-based attribution methods for Deep Neural Networks. *arXiv [cs.LG]* (2017).
32. Salvador, S. & Chan, P. Toward accurate dynamic time warping in linear time and space. *Intelligent Data Analysis* **11**, 561–580 (2007).
33. Zhang, P. *et al.* Genome-wide mapping of nucleosome positions in Saccharomyces cerevisiae in response to different nitrogen conditions. *Sci. Rep.* **6**, 33970 (2016).
34. Huisinga, K. L. & Pugh, B. F. A genome-wide housekeeping role for TFIID and a highly regulated stress-related role for SAGA in Saccharomyces cerevisiae. *Mol. Cell* **13**, 573–585 (2004).
35. Khan, A. *et al.* JASPAR 2018: update of the open-access database of transcription factor binding profiles and its web framework. *Nucleic Acids Res.* **46**, D1284 (2018).
36. Teixeira, M. C. *et al.* YEASTRACT: an upgraded database for the analysis of transcription regulatory networks in Saccharomyces cerevisiae. *Nucleic Acids Res.* **46**, D348–D353

(2018).
37. Alvarez, S. A. Chi-squared computation for association rules: preliminary results. *Boston, MA: Boston College* (2003).
38. Altschul, S. F. & Erickson, B. W. Significance of nucleotide sequence alignments: a method for random sequence permutation that preserves dinucleotide and codon usage. *Mol. Biol. Evol.* **2**, 526–538 (1985).
39. de Visser, J. A. G. M. & Krug, J. Empirical fitness landscapes and the predictability of evolution. *Nat. Rev. Genet.* **15**, 480–490 (2014).
40. Cherry, J. M. *et al.* SGD: Saccharomyces Genome Database. *Nucleic Acids Res.* **26**, 73–79 (1998).
41. de Boer, C. G. *et al.* Deciphering eukaryotic gene-regulatory logic with 100 million random promoters. *Nat. Biotechnol.* **38**, 56–65 (2020).
42. Tirosh, I. & Barkai, N. Two strategies for gene regulation by promoter nucleosomes. *Genome Res.* **18**, 1084–1091 (2008).
43. Cunningham, F. *et al.* Ensembl 2019. *Nucleic Acids Res.* **47**, D745–D751 (2019).
44. Ziemann, M., Kaspi, A. & El-Osta, A. Digital expression explorer 2: a repository of uniformly processed RNA sequencing data. *Gigascience* **8**, 1–13 (2019).
45. Thompson, D. A. *et al.* Correction: Evolutionary principles of modular gene regulation in yeasts. *Elife* **2**, e01114 (2013).
46. Houser, J. R. *et al.* An improved short-lived fluorescent protein transcriptional reporter for Saccharomyces cerevisiae. *Yeast* **29**, 519–530 (2012).
