# Supplementary material for: Deep learning suggests that gene expression is encoded in all parts of a co-evolving interacting gene regulatory structure
Source: Nat Commun. 2020 Dec 1;11:6141. doi: 10.1038/s41467-020-19921-4 (PMC7708451; doi:10.1038/s41467-020-19921-4)
Supplement: Supplementary file 3 — Reporting Summary [file 41467_2020_19921_MOESM3_ESM.pdf]

nature research

Corresponding author(s): Zelezniak Aleksej

Last updated by author(s): Oct 9, 2020

Reporting Summary

Nature Research wishes to improve the reproducibility of the work that we publish. This form provides structure for consistency and transparency in reporting. For further information on Nature Research policies, see our Editorial Policies and the Editorial Policy Checklist.

Statistics

For all statistical analyses, confirm that the following items are present in the figure legend, table legend, main text, or Methods section.

n/a	Confirmed	
☐	☒	The exact sample size (*n*) for each experimental group/condition, given as a discrete number and unit of measurement
☒	☐	A statement on whether measurements were taken from distinct samples or whether the same sample was measured repeatedly
☐	☒	The statistical test(s) used AND whether they are one- or two-sided<br>*Only common tests should be described solely by name; describe more complex techniques in the Methods section.*
☒	☐	A description of all covariates tested
☐	☒	A description of any assumptions or corrections, such as tests of normality and adjustment for multiple comparisons
☐	☒	A full description of the statistical parameters including central tendency (e.g. means) or other basic estimates (e.g. regression coefficient) AND variation (e.g. standard deviation) or associated estimates of uncertainty (e.g. confidence intervals)
☐	☒	For null hypothesis testing, the test statistic (e.g. *F*, *t*, *r*) with confidence intervals, effect sizes, degrees of freedom and *P* value noted<br>*Give P values as exact values whenever suitable.*
☒	☐	For Bayesian analysis, information on the choice of priors and Markov chain Monte Carlo settings
☒	☐	For hierarchical and complex designs, identification of the appropriate level for tests and full reporting of outcomes
☐	☒	Estimates of effect sizes (e.g. Cohen's *d*, Pearson's *r*), indicating how they were calculated

Our web collection on statistics for biologists contains articles on many of the points above.

Software and code

Policy information about availability of computer code

Data collection	Hyperopt v0.1.1; Mafft v7.407; MrBayes v3.2.6; Zorro v1.0; CD-HIT v4.8.1; Biopython v1.73; Tomtom and Meme suite v4.12; ConsensusClusteringPlus v1.48.0
Data analysis	Apache Spark v2.4; Scipy v1.1.0; Keras v2.2; Tensorflow v1.10; Scikit–learn v0.20.3, Fastdtw v0.3.2; Python v3.6; R v3.6 ; Tidyverse v1.3.0; Code for the data analysis was deposited to the Github repository and is available at https://github.com/JanZrimec/DeepExpression.

For manuscripts utilizing custom algorithms or software that are central to the research but not yet described in published literature, software must be made available to editors and reviewers. We strongly encourage code deposition in a community repository (e.g. GitHub). See the Nature Research guidelines for submitting code & software for further information.

Data

Policy information about availability of data

All manuscripts must include a data availability statement. This statement should provide the following information, where applicable:

- Accession codes, unique identifiers, or web links for publicly available datasets
- A list of figures that have associated raw data
- A description of any restrictions on data availability

Genomic data, transcript and gene boundaries were obtained from Ensembl Genomes release 41 and Ensembl release 94 (https://www.ensembl.org/), Saccharomyces Genome Database (https://www.yeastgenome.org/) and RegulonDB v10.5 database (http://regulondb.ccg.unam.mx/) (links to raw data in Tables S1-4, S2-3). RNA sequencing data was obtained from the Digital Expression Explorer V2 database (http://dee2.io/mx/), DNA sequence motifs from the Meme suite motifs databases file (http://meme-suite.org/) and additional data from the cited references (links to raw data in Supplementary table 7). The Source Data file was deposited to the Zenodo repository and is available at https://doi.org/10.5281/zenodo.3905251.

Field-specific reporting

Please select the one below that is the best fit for your research. If you are not sure, read the appropriate sections before making your selection.

[x] Life sciences [ ] Behavioural & social sciences [ ] Ecological, evolutionary & environmental sciences

For a reference copy of the document with all sections, see nature.com/documents/nr-reporting-summary-flat.pdf

Life sciences study design

All studies must disclose on these points even when the disclosure is negative.

Sample size	No sample size calculation was performed as no statistical distribution assumptions were made, deep learning models were evaluated directly on test sets without any assumption of underlying distributions.
Data exclusions	For gene expression levels, processed raw RNA sequencing Star counts were obtained from the Digital Expression Explorer V2 database (http://dee2.io/index.html) and filtered for experiments that passed quality control (QC tag in original database). Raw mRNA data were transformed to transcripts per million (TPM) counts and genes with zero mRNA output (TPM < 5) were removed (Table S1-2).
Replication	Technical duplicates were performed with experimental measurements, triplicate runs with varying random seeds were performed with deep modeling and 10 replicates were performed with shallow modeling.
Randomization	Not relevant with the design of the present study as groups were not compared.
Blinding	Not relevant with the design of the present study as trials/data were not collected, but instead publicly available RNA-Seq data was used.

Reporting for specific materials, systems and methods

We require information from authors about some types of materials, experimental systems and methods used in many studies. Here, indicate whether each material, system or method listed is relevant to your study. If you are not sure if a list item applies to your research, read the appropriate section before selecting a response.

Materials & experimental systems

n/a	Involved in the study	
[x]	[ ]	Antibodies
[x]	[ ]	Eukaryotic cell lines
[x]	[ ]	Palaeontology and archaeology
[x]	[ ]	Animals and other organisms
[x]	[ ]	Human research participants
[x]	[ ]	Clinical data
[x]	[ ]	Dual use research of concern

Methods

n/a	Involved in the study	
[x]	[ ]	ChIP-seq
[ ]	[x]	Flow cytometry
[x]	[ ]	MRI-based neuroimaging

Flow Cytometry

Plots

Confirm that:

[x] The axis labels state the marker and fluorochrome used (e.g. CD4-FITC).

[x] The axis scales are clearly visible. Include numbers along axes only for bottom left plot of group (a 'group' is an analysis of identical markers).

[ ] All plots are contour plots with outliers or pseudocolor plots.

[x] A numerical value for number of cells or percentage (with statistics) is provided.

Methodology

Sample preparation	The yeast cells were pre-cultured overnight in 96 deep well plates in 0.5 ml of minimal media with 2% glucose (see Table S6-4 for media composition) at 30°C and 300 rpm. The following day, the cultures were set up in 96 deep well plates with a starting OD600 of 0.1. After 5 h of cultivation, when the cells were in mid-exponential growth phase, the cells were diluted with water to a final OD600 of 0.02 in a total volume of 200 µl in 96 well round plates in technical duplicates.
Instrument	Guava easyCyte 8HT flow cytometry system
Software	FlowJo, version 10.6.1

Cell population abundance	No cell sorting was performed.
Gating strategy	The gating was performed using the FSC and SSC to select for yeast cells based on their size and granularity and included on average 95.5% of all measured cells. No other gating was performed as no cellular sub-populations were defined and no positive / negative staining definitions were used.

☒ Tick this box to confirm that a figure exemplifying the gating strategy is provided in the Supplementary Information.
